# ANGPTL8 is a negative regulator in pathological cardiac hypertrophy

**DOI:** 10.1038/s41419-022-05029-8

**Published:** 2022-07-18

**Authors:** Lin Hu, Jiarui Wei, Yue Zhang, Ziyuan Wang, Junming Tang, Jian Tang, Yujiu Gao, Xiaoqiao Zhang, Yifan Li, Yantong Liu, Shinan Ma, Xingrong Guo, Qiufang Zhang

**Affiliations:** 1 Department of Pharmacology; Hubei Key Laboratory of Embryonic Stem Cell Research; and Department of Geriatrics & General Medicine of Taihe Hospital, Hubei University of Medicine, Shiyan, 442000 Hubei China; 2 College of Pharmacy, Hubei University of medicine, Shiyan, 442000 Hubei China

**Keywords:** Renovascular hypertension, Cardiac hypertrophy

## Abstract

Pathological cardiac hypertrophy is an independent risk factor for heart failure and is considered a target for the treatment of heart failure. However, the mechanisms underlying pathological cardiac hypertrophy remain largely unknown. We aimed to investigate the role of angiopoietin-like protein 8 (ANGPTL8) in pathological cardiac hypertrophy. We found that serum ANGPTL8 levels were significantly increased in hypertensive patients with cardiac hypertrophy and in mice with cardiac hypertrophy induced by Ang II or TAC. Furthermore, the secretion of ANGPTL8 from the liver was increased during hypertrophic processes, which were triggered by Ang II. In the Ang II- and transverse aortic constriction (TAC)-induced mouse cardiac hypertrophy model, ANGPTL8 deficiency remarkably accelerated cardiac hypertrophy and fibrosis with deteriorating cardiac dysfunction. Accordingly, both recombinant human full-length ANGPTL8 (rANGPTL8) protein and ANGPTL8 overexpression significantly mitigated Ang II-induced cell enlargement in primary neonatal rat cardiomyocytes (NRCMs) and H9c2 cells. Mechanistically, the antihypertrophic effects of ANGPTL8 depended on inhibiting Akt and GSK-3β activation, and the Akt activator SC-79 abolished the antihypertrophic effects of rANGPTL8 in vitro. Moreover, we demonstrated that ANGPTL8 directly bound to the paired Ig-like receptor PIRB (LILRB3) by RNA-seq and immunoprecipitation-mass screening. Remarkably, the antihypertrophic effects of ANGPTL8 were largely blocked by anti-LILRB3 and siRNA-LILRB3. Our study indicated that ANGPTL8 served as a novel negative regulator of pathological cardiac hypertrophy by binding to LILRB3 (PIRB) and inhibiting Akt/GSK3β activation, suggesting that ANGPTL8 may provide synergistic effects in combination with AT1 blockers and become a therapeutic target for cardiac hypertrophy and heart failure.

## Introduction

Heart failure is still a detrimental disease with high morbidity and mortality that affects 64.3 million people worldwide [1]. In response to cardiac stress such as long-term hypertension or other stimuli, the early stage of cardiac hypertrophy plays a beneficial role in protecting the heart that maintains cardiac function [2], but prolonged cardiac hypertrophy results in gene transcription alternation, extracellular matrix (fibrosis) accumulation and cardiomyocyte size enlargement accompanied by cardiac dysfunction and ultimately leads to heart failure [3, 4]. Although many regulators have been reported to play pivotal roles in pathological hypertrophy, neurohumoral factors such as the renin-angiotensin system (RAS) and catecholamine are the main and most influential pathogenic factors. Clinically, ACEI (angiotensin converting enzyme inhibitor), AT1 (Ang II receptor) blocker and adrenergic receptor blockers are commonly used to prevent or reverse pathological cardiac hypertrophy development, but these drugs have many side effects, such as dry cough, sodium and water retention. Therefore, exploring the endogenous factors involved in cardiac protection against heart remodeling is of great significance [5].

Angiopoietin-like protein 8 (ANGPTL8) is a newly identified hormone that is mainly produced in and secreted by liver and adipose tissue in humans and participates in regulating glucose and lipid metabolism [5]. Circulating ANGPTL8 is related to metabolic parameters, oxidative stress, and inflammatory disease [6]. Serum ANGPTL8 levels were increased in patients with type 2 diabetes mellitus (T2DM) [7], positively correlated with FPG (fasting plasma glucose) and fasting C-peptide and negatively correlated with insulin resistance [8]. Recently, studies revealed that serum ANGPTL8 levels were significantly elevated in patients with coronary artery disease (CAD), thoracic aortic dissection (TAD) or essential hypertension [9]. Furthermore, an increased plasma ANGPTL8 level was closely associated with the prevalence and severity of CAD and potentially contributed to increased cardiovascular risk. Higher circulating ANGPTL8 values were associated with all-cause mortality in a 5-year follow-up study, but Leiherer et al. [10] reported that high betatrophin (ANGPTL8, upper tertile, >9.4 ng/ml) was significantly and inversely associated with cardiovascular events or death and improved the risk stratification of the SCORE algorithm in patients with stable coronary artery disease (CAD) during 8 years of follow-up. These data indicated a potential role for ANGPTL8 in cardiovascular disease. Notably, our preliminary screening found that ANGPTL8 knockout accelerated the development of pathological hypertrophy in vivo and cardiomyocyte enlargement in vitro. This evidence supports the hypothesis that ANGPTL8 may be a negative regulator in pathological cardiac hypertrophy; however, this hypothesis has still not been illustrated.

In this study, we found that ANGPTL8 ablation aggravated cardiac hypertrophy and fibrosis in TAC and Ang II infusion-induced cardiac hypertrophy models. rANGPTL8 or ANGPTL8 overexpression inhibited Ang II-induced cardiomyocyte hypertrophy in NRCMs or H9c2 cells. Mechanistically, we demonstrated that ANGPTL8 could interact with the inhibitory receptor LILRB3 directly and then block the activation of the Akt/GSK3β pathway. Collectively, our results delineated that ANGPTL8 ameliorated the development of pathological cardiac hypertrophy via the LILRB3-Akt/GSK3β signaling cascade.

## Materials and methods

### Study population and ethical statement

We recruited 200 subjects from the Affiliated Taihe Hospital of Hubei University of Medicine into this study, including 70 healthy people, 30 patients with hypertension, and 100 patients with hypertension and left ventricular hypertrophy. Subjects with hypertension were defined as having a systolic blood pressure ≥140 mmHg and/or a diastolic blood pressure ≥90 mmHg at the initial visitor or those using antihypertensive medication [11, 12]. Left ventricular hypertrophy was diagnosed by ultrasonic echocardiography and/or electrocardiogram and/or porcine magnetic resonance imaging (MRI). The control group was composed of healthy individuals without hypertension and other diseases. The exclusion criteria for all subjects, both case and controls, included a history of diabetes, hyperlipidemia, subjects with high blood glucose, fever, infectious disease, chronic hepatic diseases, autoimmune disease, arthritis, malignancies, and other severe medical diseases. Informed written consent was obtained from each patient, and the protocol was approved by the Ethics Committee of Hubei University of Medicine. All experiments in this paper obeyed the principles of the 2013 Declaration of Helsinki.

A 5 ml blood sample was obtained from all the subjects following an overnight fast. Then, plasma was prepared by centrifugation at 400 × *g* for 15 min, and the supernatants were stored at − 80 °C until analysis.

### Animals

Male C57BL/6 mice (8–10 weeks old) were obtained from the Center for Animal Experiment/Animal Biosafety-III laboratory of Hubei University of Medicine. Mice were housed under suitable temperature (23 °C) and lighting (12 h light/12 h dark cycle) with free access to water and normal chow. ANGPTL8^−/−^(ANGPTL8 KO) mice were generated in C57BL/6J mice by the CRISPR/Cas9 system using a previously described method in the Laboratory Animal Center of Sun Yat-sen University [13]. We designed three sgRNAs that were cloned into a pX330 plasmid expressing Cas9 and driven by the U6 promoter. The sgRNA target sequences used in this study are shown in a published article. ANGPTL8 sgRNA and Cas9 mRNA were coinjected into one-cell embryos, and 1 female mouse and 6 male mice were generated for the F0 generation. The genotyping of transgenic mice in the F1 generation was analyzed by DNA sequencing. All the F0 and F1 generation animals were obtained from the Laboratory Animal Center of Sun Yat-sen University, and then the F1 generation mice identified as ANGPTL8 knockout heterozygotes were transferred to the SPF Animal Experimental Center of Hubei University of Medicine for further experimental study. All animal experiments were recognized and approved by the Center’s Experimental Animal Ethics Committee.

### Cell culture and adenovirus transfection

H9c2 cells (Cell Bank of Type Culture Collection of The Chinese Academy of Sciences) were cultured with high glucose (4500 mg/l) Dulbecco’s modified Eagle’s medium (DMEM) containing 10% fetal bovine serum (FBS) at 37 °C in a CO_2_ incubator.

Primary culture of neonatal rat cardiomyocytes (NRCM) [14]. In brief, ventricular myocytes were isolated from 1~3-day-old neonatal Sprague–Dawley mice using 0.125% trypsin and 0.0625% collagenase II (pH 7.35). The cells were collected and diluted with complete DMEM with 10% FBS and preplated in a cell culture dish at 37 °C for 90–120 min to remove attached cardiac fibroblasts and endothelial cells. Then, the cells were resuspended in complete DMEM with 0.1 mM BrdU. After 24 h of culture, the medium was changed with serum-free DMEM/F12 for 12 h, and cardiomyocytes were treated with PBS or Ang II(1 μM) for 48 h to induce cardiomyocyte hypertrophy.

H9c2 cells and NRCMs were treated with recombinant human ANGPTL8 protein (rANGPTL8, c797, Nava Protein Company, 500 ng/ml) and treated with or without Ang II (1 μM) for 48 h. The Akt activator SC79 (8 µg/ml, Beyotime Biotechnology) was added to the media for 1 h before rANGPTL8 with or without Ang II administration.

For the primary mouse hepatocyte, hepatic stellate and primary hepatic Kupffer cell culture methods, see the supplementary experimental methods section.

### Lentiviral transfection

To overexpress ANGPTL8, we purchased adenoviral ANGPTL8 from Cyagen Biosciences Co. Ltd. and cloned full-length ANGPTL8 into a replication-defective lentiviral vector to generate lentiviral ANGPTL8 (G12 V). A lentiviral empty vector was used as a control. H9c2 cells were plated in culture medium on the day before transfection and reached 70–80% confluence at the time of transfection. Then, ANGPTL8 adenovirus at a concentration of 30 MOI and 5 µl of polybrene (3 µg/ml, Invitrogen, USA) auxiliary transfection reagent were added to fresh culture medium. The transfection efficiency was checked 24–48 h post-transfection, and then transfection complex-containing medium was removed gently and refilled with complete culture medium when the transfection efficiency reached more than 90%. Next, cells transfected with lentivirus were selected by the appropriate puromycin for 1 week to obtain stable cell lines for the following study.

### RNA interference assay

For the RNA interference assay, the siRNA target sequences used in this study were as follows: LILRB3 siRNA, 5’-GCAGGACAATACTGGTGTT-3’, and NC siRNA sequence, 5’-TTCTCCGAACGTGTCACGTdTdT-3’. All siRNAs were synthesized by RiboBio (Guangzhou, China) and transfected at a final concentration of 10 nM.

### Animal models and treatment

#### Ang II induced cardiac hypertrophy

A mouse cardiac hypertrophy model was induced by Ang II [1]. Briefly, the mice were implanted with an osmotic minipump (containing Ang II) under anesthesia with 1% pentobarbital sodium (50 mg/kg, i.p.), and Ang II (1.4 mg kg^−1^ per day and dissolved in 0.9% NaCl) were subcutaneously infused into mice for 4 weeks. The control animals were subjected to the same procedures as experimental animals and infused with saline (the same volume of infused Ang II).

#### Transverse aorta constriction (TAC)

A transverse aorta constriction (TAC) operation was used to establish pressure overload-triggered cardiac hypertrophy [15]. Briefly, the C57BL/6J mice were anesthetized with 1% pentobarbital sodium (50 mg/kg) by intraperitoneal injection and then placed on a heating pad in a supine position to perform endotracheal intubation to maintain artificial respiration. The aortic arch branch was exposed with a chest retractor opening of the second and third intercostal. 6.0 silk sutures were placed between the innominate and left carotid arteries, and then a 271/2 gauge blunt needle was placed parallel to the transverse aorta. The knot was tied against the needle and quickly removed to achieve a constriction of 0.4 mm in diameter. After the operation, the mouse was injected with bupivacaine (0.5 mg/100 g) subcutaneously to alleviate postoperative pain. When anesthesia gradually faded away, the endotracheal tube was removed to recover spontaneous breathing. The sham group (control group) underwent the same surgery without ligation of the aorta. After 4 weeks, TAC was confirmed by a 30 MHz Doppler probe.

#### Echocardiography

Cardiac function was evaluated by echocardiography. Briefly, echocardiography was performed using a visualsonic Vevo 2100 instrument (Visualsonics, Canada) with a 30 MHz linear array ultrasound transducer [14, 16]. M mode measurement of left ventricular (LV) inner diameter was abstained from at least three beats, and then the average was calculated. The left ventricular end-diastolic dimension (LVEDd) and left ventricular end-systolic dimension (LVESd) were measured at the largest and smallest LV areas, respectively. LV fraction shortening (LVFS) and left ventricular ejection fraction (LVEF) were calculated as follows: FS% = (LVEDd-LVESd)/LVEDd × 100%.

#### Histological analysis

Hematoxylin and eosin (H&E) staining, picric Sirius red staining (PSR), and wheat germ agglutinin (WGA) staining were performed to evaluate histopathology, fibrosis and cross-sectional area on paraffin-embedded heart sections. PSR and WGA images were analyzed by a trained hepatopathologist who was blinded to the identity of the samples according to criteria described by Kleinertal [17]. The relative myocardial interstitial fibrosis area and cross-sectional area were measured using a digital image analysis system (Image-Pro Plus, version 6.0) from images captured from PSR- and WGA-stained sections. At least 40 fields or 100 cardiomyocytes in the examined sections were profiled in each group.

Rhodamine phalloidin staining: H9c2 cells and NRCMs were fixed with paraformaldehyde (4%) for 10 min, washed with PBS, permeabilized with 0.1% Triton X-100 in PBS for 5 min, and stained with rhodamine phalloidin, followed by nuclear staining with DAPI. A quantitative digital image analysis system (Image Pro) was used to measure cell surface size. At least 50 cells were examined in each group for 5 independent experiments.

#### Immunohistochemical staining

Briefly, 5 µm serial sections were dewaxed in xylene and rehydrated through graded alcohols. Endogenous peroxidases were blocked with 3% H_2_O_2_ for 30 min, and antigens were retrieved by microwaving slides. After cooling and washing, slides were blocked with goat serum for 30 min (1:10, Zymed antibody diluent). The sections were then incubated with collagen I primary antibodies (1:100, Proteintech, Chicago, USA) at 4 °C overnight and incubated with HRP-conjugated secondary antibodies followed by the Liquid DAB Substrate Chromogen System according to the manufacturer’s instructions. At least 10 fields in the examined heart sections were measured in each group by Image-Pro Plus 6.0.

#### Immunofluorescence staining

To detect ANGTPL8 expression, the heart and liver tissues were fixed in paraformaldehyde (4%) for 12 h, embedded in paraffin, and then serially sectioned transversely at 5 μm thickness. Subsequently, sections were heated using the pressure cooker method for antigen retrieval and incubated with anti-ANGPTL8 antibody (1:100, Sigma, St. Louis, Missouri, USA) overnight at 4 °C. Then, the slices were washed and stained with a fluorescence-conjugated secondary antibody (Cell Signaling, Alexa Flur 594). The slices were also stained with FITC-WGA-lectin and DAPI to label the cell membrane and cell nucleus, respectively. Immunofluorescence was analyzed with confocal microscopy (Olympus Corporation), and the fluorescence intensity of at least 10 fields in examined heart sections was measured in each group by Image-Pro Plus 6.0.

#### Cytokine measurements

The ANGPTL8 concentration in plasma was determined with ELISA using a human ANGPTL8 ELISA kit (Elabscience Biotechnology Co., Ltd. Wuhan, China) [18]. The intra-assay and interassay variations were <10% and <15%, respectively. The linear range of the assay was 0–100 pg/ml. The assay has high sensitivity and excellent specificity for the detection of ANGPTL8 with no significant cross-reactivity or interference.

#### Co-immunoprecipitation

FLAG-mANGPTL8 and Pe-CFP-PIRB were transfected into 293-T cells using Lipo8000™ Transfection Reagent. After 24 h, 293-T cells were collected and lysed in ice-cold IP buffer (containing 50 mM Tris-HCl, pH 7.5, 150 mM NaCl, 1% Nonidet P40 (Np40), proteinase inhibitor (Roche), 0.1% SDS-Na) for 30 min. The lysates were sonicated and centrifuged at 13,000 × *g* for 15 min at 4 °C. The supernatants were collected and incubated with anti-FLAG-M2 agarose beads (Sigma) overnight at 4 °C. The beads were rinsed with ice-cold IP buffer three times and then heated at 95 °C in loading buffer, followed by western blot analyses. Images of the precipitated proteins were captured by a Bio-Rad imaging system and analyzed by Image J.

#### Quantitative real-time polymerase chain reaction (qRT–PCR)

Total RNA was extracted from cells using TRIzol (Invitrogen). Subsequently, cDNA was synthesized using the Superscript II kit for RT–PCR (TIANGEN Biotech). qRT–PCR was conducted in a 10 μl reaction mixture containing 50 nM forward and reverse primers, 1 × SYBR Green reaction mix (QIAGEN Bioinformatics) and 3.2 nM template. The sequences of primers for quantitative real-time PCR are listed in Supplementary Table 3. β-actin was used as a control, and the delta–delta Ct method was used for data analysis. Five biological replicates were performed for the experiments.

#### Western blotting

Total protein was extracted from heart tissues and NRCMs or H9c2 cells. The protein concentration was determined by the BCA method. Forty micrograms of total protein was loaded and separated by 10% SDS–PAGE and transferred to PVDF membranes. After blocking with 5% skimmed milk, the membranes were incubated with antibodies against Akt (Ser473), p-Akt, Collagen I, MYH7 (β-MHC) (1:1000, Proteintech, Chicago, USA), GSK3β, p-GSK3β (1:1000, Cell Signaling Technology, Boston, USA), ANGPTL8 (1:1000, Sigma, St. Louis, Missouri, USA), LILRB3 (ABclonal Technology Co., Ltd. Chicago, USA), β-actin (1:2000, Beyotime Biotechnology, Shanghai, China), BNP (1:1000, Dallas, USA) and β-tubulin (1:1000, Absin, Shanghai, China) at 4 °C overnight. After washing 3 times with TBST, the membranes were incubated with horseradish peroxidase (HRP)-conjugated secondary antibody (1:1000, Beyotime Biotechnology, Shanghai, China) at RT for 1 h. Then, the membranes were washed 3 times with TBST and imaged with a gel imaging system (BIO-RAD, California, USA). Three independent experiments were performed.

#### Statistical analysis

The data are expressed as the mean ± SD (standard deviation), and all data were analyzed with SPSS 22.0 software. The two samples were compared by 2-tailed Student’s *t* test. One-way ANOVA was used to compare the means of >2 groups when the data met a normal distribution. The least significant difference (LSD) or Tamhane’s T2 test was applied between groups for data meeting homogeneity of variance or showing heteroscedasticity. *P* < 0.05 was considered statistically significant.

## Results

### The ANGPTL8 level was increased in subjects with cardiac hypertrophy

**Fig. 1:**
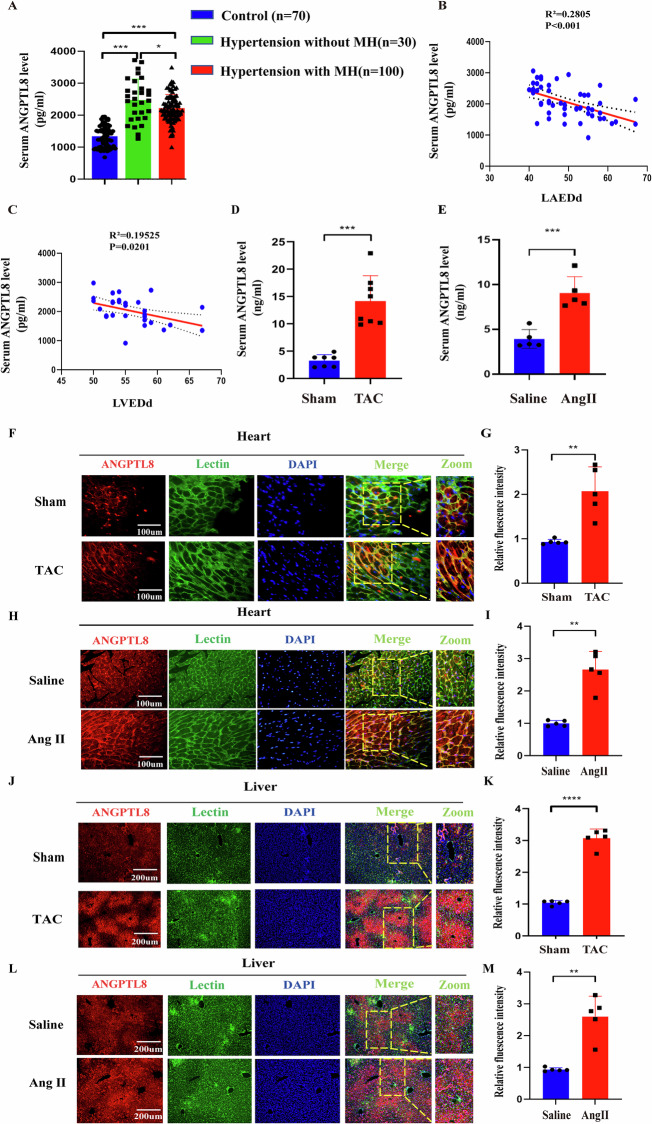
Serum ANGPTL8 was increased in patients and animal models with cardiac hypertrophy. **A** Serum levels of ANGPTL8 in patients with hypertension (*n* = 30), patients with hypertension and myocardial hypertrophy (MH) (*n* = 100) and healthy people (*n* = 70). **B**, **C** The correlation between serum ANGPTL8 level and left atrium diastolic diameter (LAEDd) (*n* = 39) or left ventricular end diastolic diameter (LVEDd) (*n* = 54) in the subjects with hypertensive patients and myocardial hypertrophy. Serum ANGPTL8 levels in mice subjected to TAC for 4 weeks (**D**, *n* = 7 mice per group) and Ang II-infused mice (**E**, *n* = 5 mice per group) were detected by ELISA (***P < *0.001*). **F** Representative image of immunofluorescence with an anti-ANGPTL8 antibody in slices from TAC and sham mice (*n* = 5 mice per group, scale bar, 100 μm). **G** Quantitative results of the relative fluorescence intensity of ANGPTL8 in the indicated groups (*n* = 5 mice per group, scale bar, 100 μm, **P < 0.01). **H**, **I** Representative image and quantitative results of immunofluorescence with an anti-ANGPTL8 antibody in the indicated groups (*n* = 5 mice per group, scale bar, 100 μm, **P < 0.01). **J**–**M** Representative immunofluorescence images and quantitative results of ANGPTL8 in slices from the indicated mouse livers (*n* = 5 mice for the sham or TAC group; *n* = 5 mice for the saline or Ang II group, scale bar, 200 μm, **P < 0.01, ****P < 0.0001).

To evaluate whether the circulating ANGPTL8 level was associated with pathological cardiac hypertrophy, the serum ANGPTL8 concentration was measured in patients with hypertension and healthy people. Serum ANGPTL8 was increased in subjects with hypertension and myocardial hypertrophy (MH) compared with healthy people (2108 ± 134.86 *vs* 1218 ± 156.74 ng/ml) but was lower than that in subjects with hypertension without complications (2108 ± 134.68 *vs* 2516 ± 289.21 ng/ml) (Supplementary Table 1 and Fig. 1A). Moreover, serum ANGPTL8 levels were negatively correlated with LAEDd and LVEDd in patients with hypertension and MH (Fig. 1B, C). In parallel, in the experimental mouse model of TAC- and Ang II-induced cardiac hypertrophy, serum ANGPTL8 levels were also dramatically increased (Fig. 1D, E).

To explore the source of ANGPLT8 in the serum, we first performed human single-cell sequencing and found that there was no ANGPTL8 expression on cardiomyocytes (Supplementary Fig. S1A, B). Then, we further identified that no ANGPTL8 mRNA was expressed in mouse hearts after 4 weeks of Ang II treatment (Supplementary Fig. S1C). In addition, ANGTPL8 was not expressed in NRCMs or neonatal rat cardiac fibroblasts (NFS) stimulated with Ang II for 48 h (Supplementary Fig. S1C). We also detected whether ANGTPL8 expression was altered in other organs in a mouse model of cardiac hypertrophy. Our results indicated that ANGPTL8 mRNA was significantly increased in liver tissue and was mainly expressed in primary hepatocytes, with little expression in Kupffer cells, but not in primary hepatic stellate cells (Supplementary Figs. S1D and S3A–C), and no alterations in other organ tissues, including lung, spleen, brain, stomach and skeleton muscle (Supplementary Fig. S2A–R). Furthermore, ANGPTL8 mRNA was upregulated by Ang II in primary mouse hepatocytes (Supplementary Fig. S1E, F) and HepG2 cells in a time- and dose-dependent manner (Supplementary Fig. S1H, I). More importantly, the increased ANGPTL8 expression was primarily localized to the cytoplasm of hepatocytes but in heart tissue, mainly on the cell membrane of cardiomyocytes (Fig. 1F–M). Furthermore, ANGPTL8 levels were consistently increased in Ang II-stimulated primary hepatocyte culture medium (Supplementary Fig. S1G). Thus, these data supported that the increased ANGPTL8 in the serum of the pathological cardiac hypertrophy model was derived from the liver stimulated by Ang II.

### ANGPTL8 deficiency accelerated cardiac hypertrophy and fibrosis induced by Ang II

**Fig. 2:**
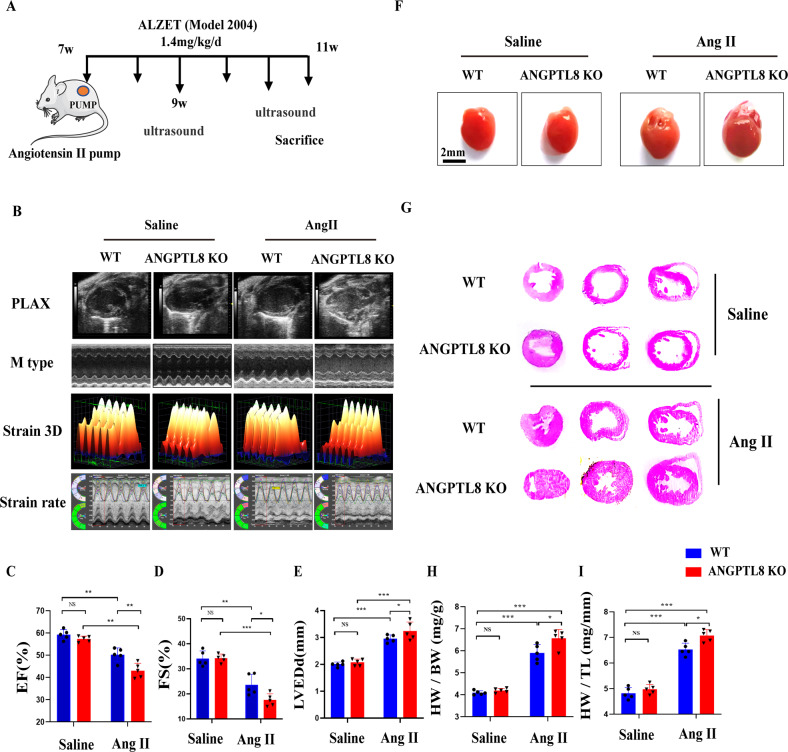
ANGPTL8 deficiency accelerated cardiac hypertrophy induced by Ang II infusion. **A** Cardiac hypertrophy in mice was induced by Ang II infusion for 4 weeks. **B** Echocardiographic assessments of cardiac function in WT and ANGPTL8-KO mice at 4 weeks of Ang II infusion. PLAX = Long axis of the heart, M model (M type), Three-dimensional deformation ability (Strain 3D&strain rate), *n* = 4~6 per group. **C**–**E** Ejection fraction (EF%), fraction shortening (FS%) and left ventricular end-diastolic dimension (LVEDd) were assessed by echocardiography in WT and ANGPTL8-KO mice at 4 weeks after saline or Ang II infusion (*n* = 5, NS no significant difference, *P < 0.05, **P < 0.01, ***P < 0.001). **F** Representative images of the gross cardiac morphology used for HW/BW and HW/HL calculation (scale bar, 2 mm) (*n* = 5). **G** Representative images of transverse sections stained with H&E (scale bar, 0.5 cm, *n* = 5). **H**, **I** Bar graphs showing quantitative data for HW/BW or HW/TL (*n* = 5 per group, NS no significant difference, *P < 0.05, ***P < 0.001).

**Fig. 3:**
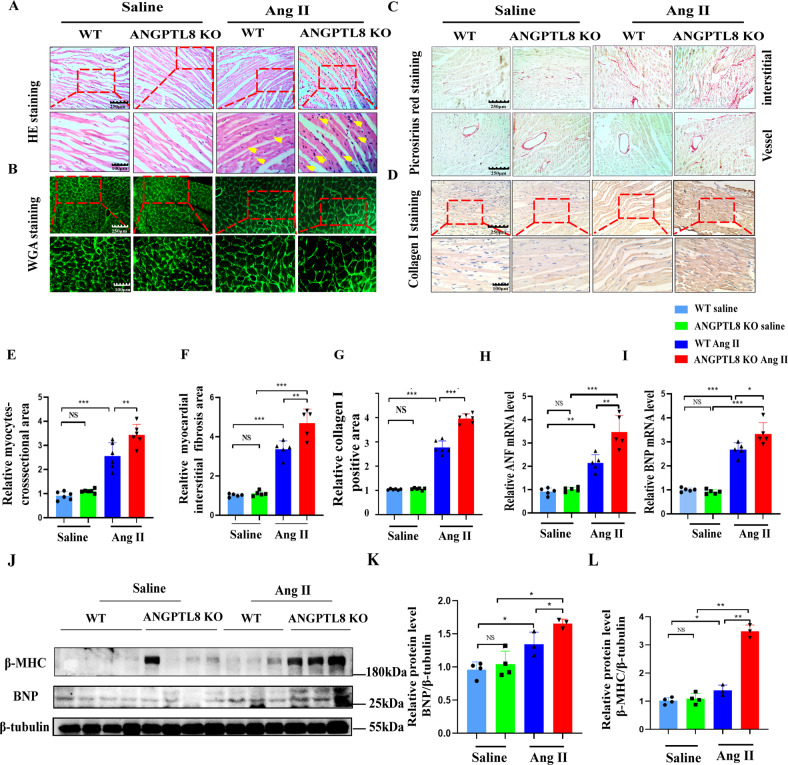
ANGPTL8KO enhanced hypertrophic and fibrotic marker expression after Ang II infusion for 4 weeks. Representative images of H&E staining (**A**), WGA staining (**B**), picrosirius red (PSR) staining (**C**) and collagen I staining (**D**) of slices from transverse sections of the left ventricles from the mouse hearts in the indicated groups (*n* = 5 mice per group, scale bars, 250 μm and 100 μm, respectively). Quantification of the average myocardial interstitial area (**E**) and myocyte cross-sectional areas (**F**) in WT and ANGPTL8-KO mice at 4 weeks (**E** *n* > 40 fields and **F** *n* = 100 cardiomyocytes from 5~6 mice in each group, NS no significant difference, **P < 0.01, ***P < 0.001). **G** Quantitative results of the collagen I-positive area from collagen I immunochemical staining in WT and ANGPTL8-KO mice (*n* = 5 mice in each group, **P < 0.01). **H**, **I** mRNA levels of ANF and BNP were analyzed by qRT–PCR from WT and ANGPTL8-KO mouse hearts (*n* = 5 mice in each group). **J** Representative Western blots of β-MHC and BNP in cardiac tissue from WT and ANGPTL8-KO mice (*n* = 3). **K**, **L** Quantitative statistical results of β-MHC and BNP protein levels (*n* = 3, NS no significant difference, *P < 0.05, **P < 0.01, ***P < 0.001).

To explore the role of ANGPTL8 in the development of pathological cardiac hypertrophy, the deficiency of ANGPTL8 protein in ANGPTL8 global knockout mice was confirmed in the liver (Supplementary Fig. S4A). ANGPTL8 KO mice did not show apparent abnormalities at baseline (Fig. 2B–I). After 4 weeks of Ang II infusion (Fig. 2A), echocardiographic assessment of left ventricular function showed that ANGPTL8 KO accelerated cardiac dysfunction (Fig. 2B), as evidenced by decreases in fraction shortening (FS%) and ejection fractions (EF%) and increases in left ventricular end-diastolic dimension (LVEDd) (Fig. 2C, E). Furthermore, the ratios of heart weight/body weight (HW/BW) and heart weight/tibia length (HW/TL) were markedly increased in ANGPTL8 KO mice compared with WT mice (Fig. 2H, I & Supplementary Table 2 model 1). ANGPTL8 KO mice also displayed a larger gross size of the heart and larger cardiomyocyte cross area in response to Ang II than WT mice (Fig. 2F, G). In addition, H&E, wheat germ agglutinin (WGA), picrosirius red (PSR) and collagen I staining showed that ANGPTL8 KO enhanced Ang II-induced hypertrophic remodeling and fibrosis of the myocardial tissue compared with WT (Fig. 3A–G). Consistently, the expression of the hypertrophic and fibrosis markers BNP (brain natriuretic peptide), β-MHC (β-myosin heavy chain), and ANF (atrial natriuretic factor) was significantly increased in ANGPTL8 KO mice compared with WT mice (Fig. 3H–L). Altogether, these findings indicated that ANGPTL8 KO dramatically aggravated cardiac hypertrophy and fibrosis induced by Ang II.

### ANGPTL8 ameliorated cardiomyocyte hypertrophy induced by Ang II in vitro

**Fig. 4:**
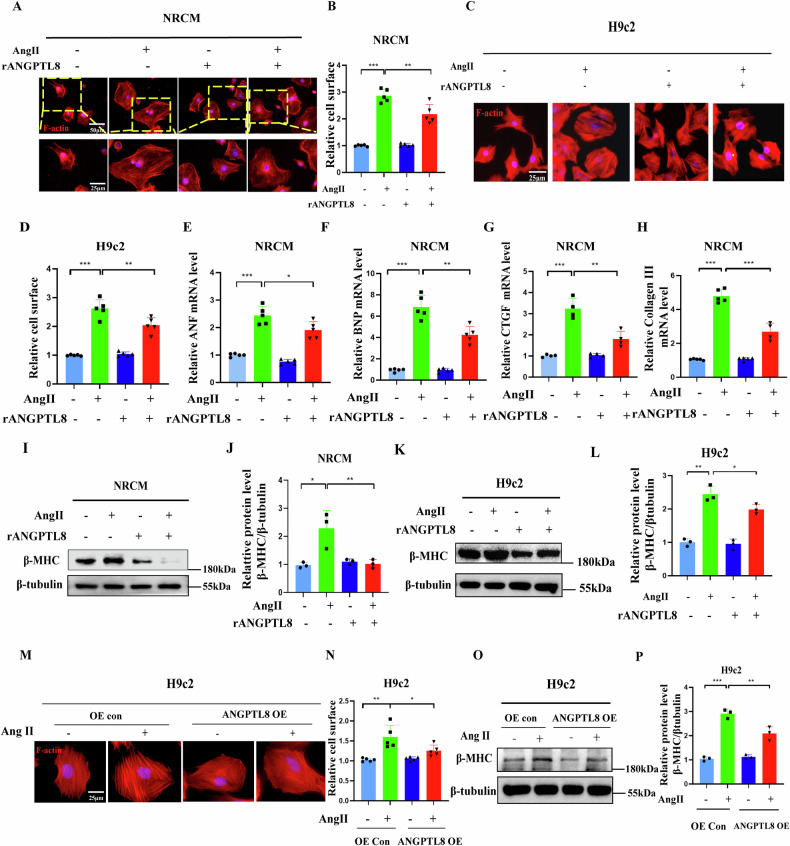
ANGPTL8 ameliorated cardiomyocyte hypertrophy induced by Ang II in vitro. Primary NRCM or H9c2 cells were treated with recombinant human full-length ANGPTL8 protein (rANGPTL8, 500 ng/ml) and Ang II (1 µM) for 48 h. **A** Representative microscopic images of NRCMs stained with rhodamine phalloidin in the indicated groups (scale bar, 50 μm and 25 μm, respectively). **B** Quantitative results of the cell surface area of cardiomyocytes in the indicated groups (*n* > 50 cells per group, 5 independent experiments, **P < 0.01, ***P < 0.001). **C** Representative microscopic images of H9c2 cells in the indicated groups (scale bar, 25 μm). **D** Quantitative results of the cell surface area of H9c2 cells in the indicated groups (*n* > 50 cells per group, 5 independent experiments, **P < 0.01, ***P < 0.001). **E**–**H** Quantification of the mRNA levels of ANF, BNP, collagen III and CTGF in NRCM in the indicated groups (*n* = 5 per group, **P < 0.01, ***P < 0.001). **I**–**L** Representative western blots and quantification of β-MHC in NRCM and H9c2 cells in the indicated groups (*n* = 3 per group, *P < 0.05, **P < 0.01). H9c2 cells were infected with the indicated lentivirus of ANGPTL8 for 24 h and then treated with Ang II (1 µM) for 48 h. **M**, **N** Representative microscopic images and quantitative results of the cell surface area of H9c2 cells in the indicated groups (*n* > 50 cells per group, 5 independent experiments, scale bar, 25 μm). **O**, **P** Representative Western blots and quantification of H9c2 cells in the indicated groups (*n* = 3 per group). (OE overexpression *P < 0.05, **P < 0.01, ***P < 0.001).

To evaluate the specific role of ANGPTL8 in cardiomyocyte hypertrophy in vitro. First, recombinant human full-length ANGPTL8 protein (rANGPTL8, 500 ng/ml) was used to treat cardiomyocytes (NRCM and H9c2) stimulated with or without Ang II (1 µM) for 48 h. rANGPTL8 had no apparent effects on the cellular surface of cardiomyocytes (NRCM and H9c2) without adding Ang II but strongly decreased cardiomyocyte enlargement induced by Ang II (Fig. 4A–D). Meanwhile, rANGPTL8 reduced the mRNA levels of both the hypertrophic markers ANF, BNP, and β-MHC (Fig. 4E, F, I–L) and the fibrotic markers CTGF (connective tissue growth factor) and collagen III (Fig. 4G, H) in Ang II-treated cells. In addition, rANGPTL8 also alleviated the hypertrophic morphology and beating frequency of NRCMs stimulated with Ang II (Supplementary Fig. S5A, B). Consistently, ANGPTL8 overexpression alleviated cardiac hypertrophy induced by Ang II, leading to a damper response to Ang II-induced hypertrophy based on the analysis of cell surface area and the expression level of β-MHC (Fig. 4M, P).

ANGPTL8 was secreted from the liver, so we treated primary hepatocytes with Ang II for 12 h and then collected the culture medium to treat H9c2 cells exposed to Ang II (1 µM). The results showed that the culture medium collected from primary hepatocytes of WT mice treated with Ang II reduced the cellular surface of H9c2 and ANF and BNP mRNA expression in NRCMs (Supplementary Fig. S4B–E). In contrast, those from ANGPTL8 KO mice had no effects on hypertrophic markers (ANF, BNP) (Supplementary Fig. S4F, G). Therefore, these results indicated that exogenous ANGPTL8 represses cardiac hypertrophy directly and that ANGPTL8 overexpression inhibits cardiac hypertrophy induced by Ang II.

### ANTPTL8 knockout aggravated pressure overload-induced cardiac hypertrophy

**Fig. 5:**
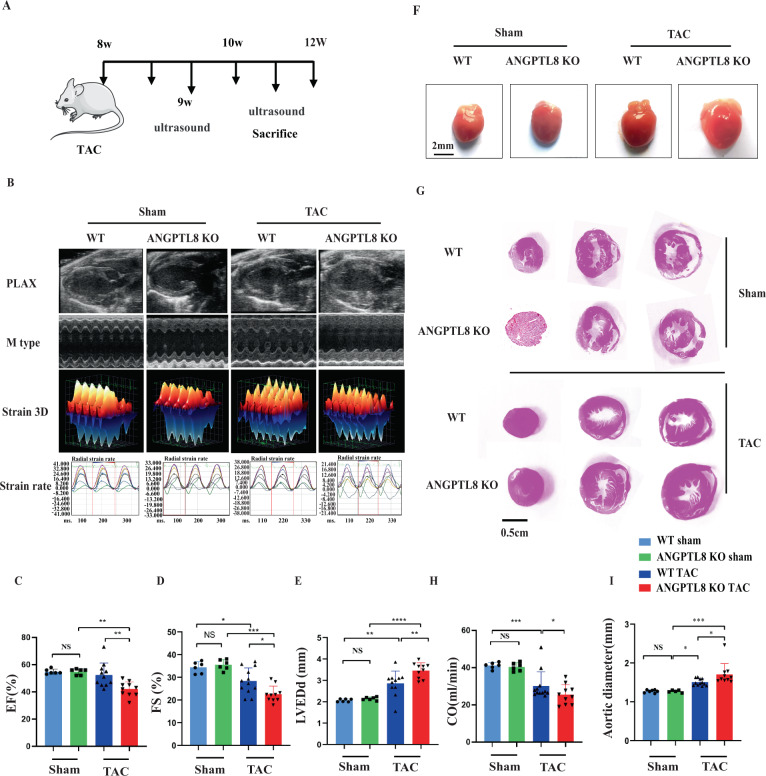
ANTPTL8 knockout aggravated pressure overload-induced cardiac hypertrophy. A pressure overload-induced cardiac hypertrophy model was established by transverse aorta constriction (TAC) for 4 weeks. **A** Schematic diagram depicting TAC. **B** Echocardiographic detection of cardiac function in mice with myocardial hypertrophy at 4 weeks after TAC surgery, long axis of the heart (PLAX), M model (M type), and three-dimensional deformation ability (Strain3D & strain rate). **C–E**, **H**, **I** Echocardiographic assessments of EF%, FS%, LVEDd, cardiac output (CO) and aortic diameter (AD) in WT and ANGPTL8-KO mice at 4 weeks after sham (*n* = 6 mice) or TAC surgery (*n* = 9~10) (NS no statistical significance, *P < 0.05, **P < 0.01, ***P < 0.001, ****P < 0.0001). **F** Representative images of the gross cardiac morphology of WT and ANGPTL8-KO mice in the indicated groups. **G** Representative images of H&E staining from transverse sections of the left ventricles from the indicated groups (*n* = 5 mice for each group, scale bar, 0.5 cm).

**Fig. 6:**
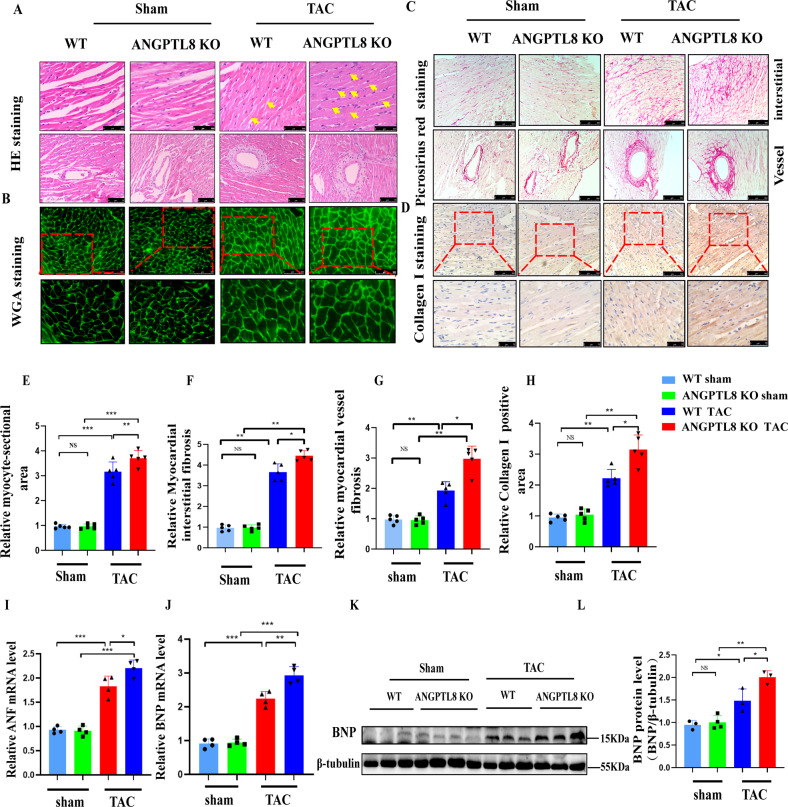
ANTPTL8 knockout enhanced hypertrophic and fibrotic features in the heart at 4 weeks after TAC. Representative images of H&E **A** (scale bar, 500 μm (up) and 75 μm (down), respectively) and WGA staining **B** (scale bar, 500 μm and 75 μm, respectively) in the indicated groups (*n* = 5 mice in each group). **C** Picrosirius red-stained transverse sections of the left ventricles from the indicated groups (scale bar, 500 μm, *n* = 6 mice in each group). **D** Representative immunohistochemical images for collagen I in mouse tissue with sham and TAC surgery in the indicated groups (scale bar, 500 μm and 75 μm, respectively, *n* = 6 mice in each group). Quantification of the myocyte-sectional area (**E**), myocardial interstitial fibrosis (**F**), myocardial vessel fibrosis (**G**) and collagen I-positive area (**H**) of slices from WT and ANGPTL8-KO mice in the indicated groups (*n* = 5 mice in each group). **I**, **J** qRT–PCR was performed to determine the mRNA levels of ANF and BNP in the indicated groups (*n* = 4 mice in each group). Representative Western blots (**K**) and quantification (**L**) of the protein levels of BNP in cardiac tissue from WT and ANGPTL8-KO mice in the indicated groups (*n* = 4 mice in each group), *P < 0.05, **P < 0.01, ***P < 0.001.

To verify whether the antihypertrophic effect of ANGPTL8 was limited to Ang II-induced cardiac hypertrophy, we adopted overload pressure-induced cardiac hypertrophy by TAC. ANGPTL8 KO mice showed no difference from WT mice at baseline, but after 4 weeks of TAC surgery (Fig. 5A), ANGPTL8 KO exacerbated cardiac dilation and dysfunction (Fig. 5B), as evidenced by the increases in LVEDd and aortic diameter (AD) and the decreases in FS%, EF% and cardiac output (CO) (Fig. 5C–E, H, I). ANGPTL8 depletion markedly increased the hypertrophic response to pressure overload (increased HW/BW, HW/TL (Supplementary Table 2 model 2)) and cardiac appearance (Fig. 5F, G). However, BW and liver weight (LW)/BW were not significantly different between WT and ANGPTL8 KO mice subjected to Ang II or saline infusion and the TAC or sham group (Supplementary Fig. S6A–D). Consistently, H&E, WGA, PSR and immunohistochemical staining showed that ANGPTL8 KO enhanced TAC-induced hypertrophic features and aggravated perivascular and interstitial fibrosis (Fig. 6A–H). In addition, qPCR results showed that the hypertrophic genes ANF and BNP were upregulated in ANGPTL8 KO mice compared with WT mice (Fig. 6I–L). Collectively, ANGPTL8 KO also promoted TAC-induced cardiac hypertrophy.

### ANGPTL8 regulated cardiac hypertrophy via the Akt/GSK-3β pathway

**Fig. 7:**
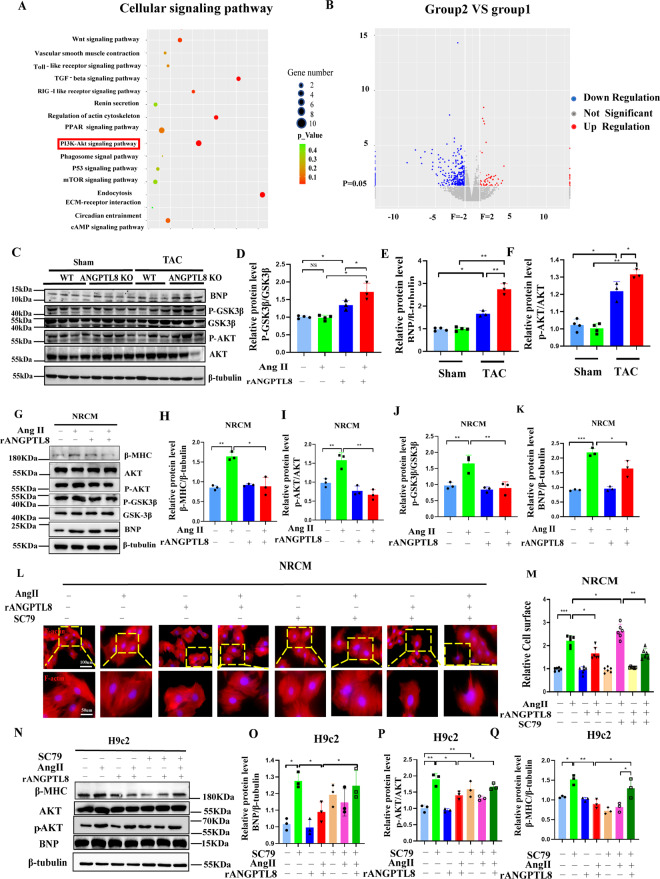
Effects of ANGPTL8 on the Akt/GSK-3β signaling pathway. **A** Kyoto Encyclopedia of Genes and Genomes pathway enrichment analysis of the identified differentially expressed genes based on RNA-seq data set from the hearts of wild-type (WT) or ANGPTL8 KO mice at 4 weeks after Ang II infusion (*n* = 3 in each group). **B** Volcano plot of relative RNA expression from the hearts of WT mice infused with Ang II for 4 weeks compared to ANGPTL8 KO mice. Genes in the upper left and right quadrants are significantly differentially expressed (microarray, *n* = 2 per group). **C**–**F** Western blots and quantification of BNP and total and phosphorylated levels of GSK-3β and Akt proteins in cardiac tissues from WT and ANGPTL8-KO mice subjected to sham (*n* = 4) or TAC (*n* = 3). **G**–**K** The effect of rANGPTL8 on NRCM hypertrophy induced by Ang II and Western blotting was used to detect the hypertrophic markers β-MHC and BNP and the total and phosphorylated protein levels of GSK-3β and Akt. Representative western blots (**G**) and quantitative results (**H–K**) of these hypertrophic markers and signaling proteins (*n* = 3 in each group). **L** Microscopy images of NRCM hypertrophy treated with rANGPTL8 or the Akt activator SC79 (scale bars, 100 μm and 50 μm, respectively). **M** Quantitative data of the cell surface of NRCMs in the indicated groups (*n* > 50 cells in each group, 6 independent experiments). **N**–**Q** Representative western blots and quantitative results of BNP, β-MHC, total Akt and phosphorylated Akt in H9c2 cells hypertrophy induced by Ang II (1 µM) in the presence of SC79 and/or rANGPTL8 (*n* = 3 in each group). *P < 0.05, **P < 0.01.

To further elucidate the underlying mechanism of ANGPTL8 against pathological cardiac hypertrophy, we performed Kyoto Encyclopedia of Genes and Genomes pathway enrichment analysis based on an RNA-seq data set from ANGPTL8-KO and WT hearts treated with Ang II (Fig. 7A, B). The PI3K/Akt signaling pathway was obviously different between ANGPTL8 KO and WT mice. Moreover, given that Akt signaling is a well-accepted key hub in pathological cardiac hypertrophy, we first assessed the potential involvement of Akt and downstream signaling in the antihypertrophic effect of ANGPTL8. ANGPTL8 KO promoted the phosphorylation of Akt (p-Akt) and its downstream substrate glycogen synthase kinase 3β (GSK-3β) in TAC-induced hypertrophic hearts (Fig. 7C–F). In contrast, rANGPTL8 markedly decreased the ratios of p-Akt/Akt and p-GSK-3β/GSK-3β in NRCMs induced by Ang II (Fig. 7G–K). The Akt activator SC-79 (8 µg/ml) enlarged cardiomyocyte size (Fig. 7L, M) and enhanced the expression levels of hypertrophic genes (Fig. 7N–Q). Notably, the Akt activator abolished the antihypertrophic effects of rANGPTL8 (Fig. 7L–Q). All the results supported that ANGPTL8 regulated cardiac hypertrophy via the Akt/GSK-3β pathway.

### ANGPTL8 directly interacted with LILRB3

**Fig. 8:**
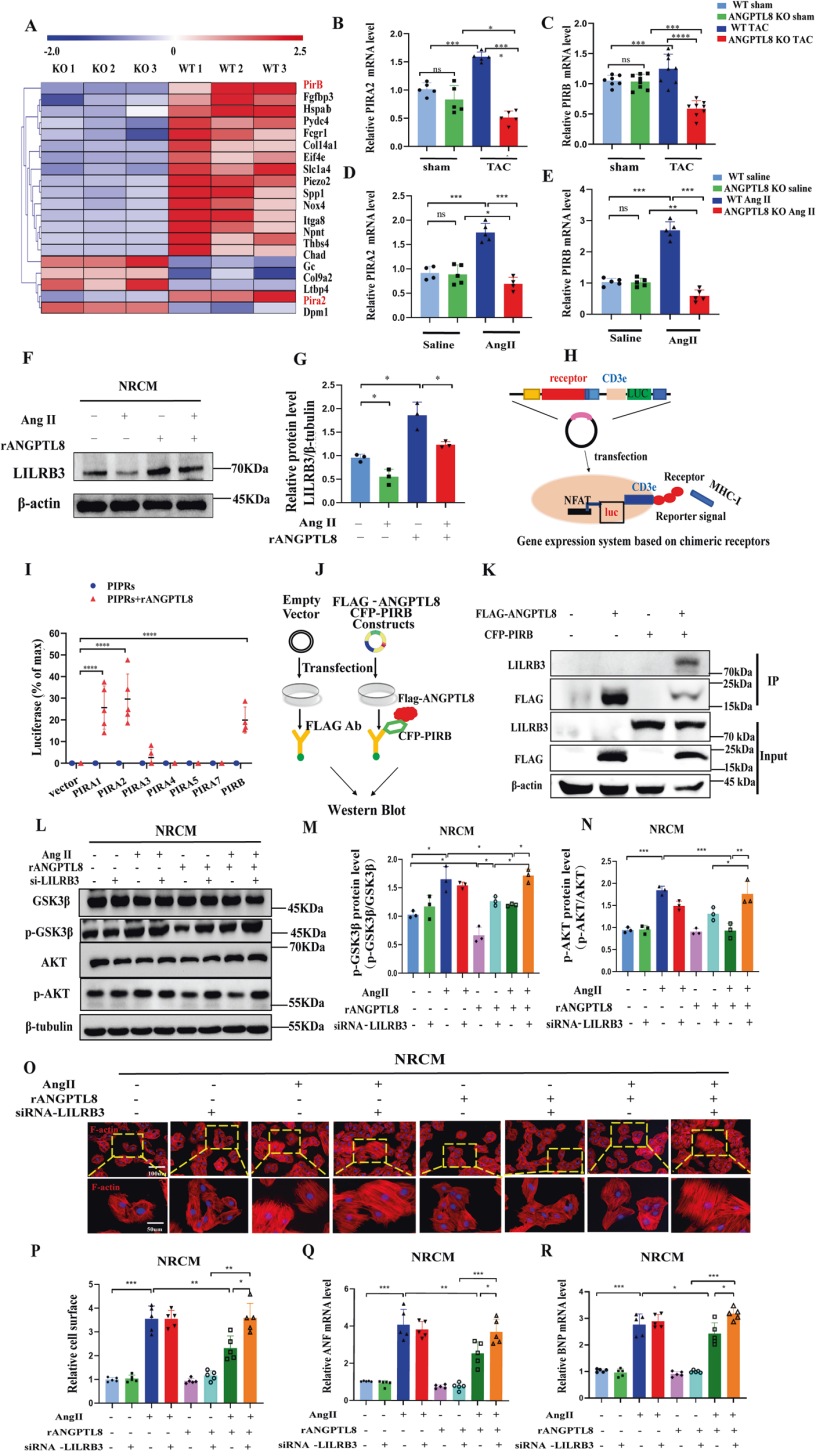
ANGPTL8 attenuated cardiomyocyte hypertrophy by interacting with LILRB3. **A** Heatmap analysis of differentially expressed genes is presented. **B**, **C** The mRNA levels of PIRA2 and PIRB were determined by qRT–PCR in WT or ANGPTL8-KO mice at 4 weeks after sham or TAC surgery (*n* = 5–8 in each group). **D**, **E** The mRNA levels of PIRA2 and PIRB were detected by qPCR in WT or ANGPTL8-KO mice at 4 weeks after saline or Ang II infusion (*n* = 4–5 in each group). **F**, **G** Representative western blots and quantification of LILRB3 in NRCM-treated rANGPTL8 and/or Ang II cells (repeated *n* = 3 per group). **H** Models of the gene expression system based on chimeric receptors. **I** A luciferase reporter system detected the interaction between ANGPTL8 and PIRs (*n* = 4–5). **J** A schematic diagram of the experimental procedure was used to identify potential targets of ANGPTL8. **K** Representative Western blots performed with Flag or LILRB3 antibody after co-immunoprecipitation (IP) of ANGPTL8 or LILRB3 from HEK-T293 cell lysates using Flag antibody or LILRB3 antibody, respectively (*n* = 3). **L**, **N** Representative western blots and quantification of total and phosphorylated Akt and GSK-3β in the presence of rANGPTL8 and/or Ang II after LILRB3 siRNA transfection (*n* = 3). **O**, **P** Representative microscopic images of rhodamine phalloidin staining and quantitative results of the cell surface of NRCM treated with Ang II and/or rANGPTL8 in the presence of siRNA-LILRB3 for 48 h (*n* > 50 cells in each group, 5 independent experiments, scale bars, 100 μm and 50 μm, respectively). **Q**, **R** The mRNA levels of the hypertrophic genes ANF and BNP were detected by qPCR in the indicated groups (*n* = 5). *P < 0.05, **P < 0.01, ***P < 0.001.

As a secreting hormone, ANGPTL8 is unable to penetrate cell membranes to regulate cardiac hypertrophy, so we explored the potential ANGPTL8 receptors on the cell membranes of cardiomyocytes. First, RNA-seq results demonstrated higher levels of paired Ig-like receptor (PIR) PIRA2 and PIRB in the hypertrophic hearts of WT mice than in those of ANGPTL8 KO mice treated with Ang II (Fig. 8A). The PIRs in rodents or leukocyte Ig-like receptors (LILRs) in humans are known as specific receptors of ANGPTLs. Previous studies have demonstrated that the human or rat immune inhibitory receptor leukocyte immunoglobulin-like receptor B3 (LILRB3) and its mouse ortholog paired immunoglobulin-like receptor (PIRB) are receptors for several angiopoietin-like proteins. Meanwhile, PIRB, as an inhibitory receptor, contributes to damaged nerve regeneration and inhibits neurite outgrowth. However, the function of PIRAs and PIRB in the mouse heart is not clear, and the ligands of LILRB3 have not yet been reported. Our results showed that the mRNA levels of PIRA2 and PIRB were upregulated in hypertrophic cardiomyocytes induced by TAC or Ang II in WT mice compared with ANGPTL8 KO mice, but there was no significant difference between WT and ANGPTL8 KO mice in the control groups (Fig. 8B–E). Consistently, rANGPTL8 enhanced LILRB3 expression in the NRCM of an in vitro hypertrophic model. In particular, the group treated with rANGPTL8 alone had higher levels of LILRB3 than the rANGPTL8+Ang II group (Fig. 8F, G). Then, we used a luciferase reporter system to analyze the relationship between ANGPTL8 and PIRs and found that ANGPTL8 could interact with PIRA1, PIRA2 and PIRB but not with PIRA3, PIRA4 or PIRA5 (Fig. 8H, I). Co-IP assays showed that ANGPTL8 could bind to PIRB directly (Fig. 8K). We further explored whether LILRB3 was involved in antihypertrophy and inhibiting Akt/GSK3β signaling of ANGPTL8. As expected, a LILRB3 antibody (anti-LILRB3) and LILRB3 siRNA abolished the protective effect of ANGPTL8 on Ang II-induced NRCM hypertrophy (Fig. 8O, R, Supplementary Fig. S5C, D) and significantly reversed the effect of rANGPTL8 inhibition of Akt/GSK3β protein phosphorylation induced by Ang II (Fig. 8L, N). Taken together, these results indicated that secreted ANGPTL8 ameliorated cardiac hypertrophy by binding to the membrane receptor PIRB (LILRB3) to suppress Akt/GSK3β activity (Supplementary Fig. S7).

## Discussion

ANGPTL8 is known as betatrophin, lipasin and TD26 and shows a regulatory function in lipid and glucose metabolism [19], which is involved in metabolic diseases such as diabetes, obesity [12, 13] and metabolic syndrome [20]. Circulating ANGPTL8 was not only enhanced in patients with infectious disease [21], but ANGPTL8 mRNA was also expressed at high levels in mice treated with LPS, acting as a “brake” for inflammation [9, 17, 22]. Moreover, serum ANGPTL8 levels were increased in patients with hypertension [11] and atherosclerotic cardiovascular disease [21, 23]. These studies mainly focused on lipid and glucose metabolism and their relationship with cardio-metabolic risk factors, but there was no research reporting the influence of ANGPTL8 on pathological cardiac hypertrophy. Herein, our clinical data demonstrated that ANGPTL8 levels were elevated in patients with hypertension complicated with myocardial hypertrophy and negatively associated with the thickness of the ventricular wall. In an animal study, ANGPTL8 knockout accelerated Ang II- and TAC-induced cardiac hypertrophy, fibrosis and dysfunction. Moreover, rANGPTL8 and ANGPTL8 overexpression attenuated Ang II-induced cardiomyocyte enlargement. Collectively, these results demonstrated that ANGPTL8, acting as a novel regulator, played a protective role in cardiac hypertrophy.

Many studies have found that serum ANGTPL8 levels are elevated in type 2 diabetes patients. Diabetes mellitus is characterized by insulin resistance and hyperglycemia, the latter of which can induce cardiac damage and exacerbate the hypertrophic response. However, in the early stages of diabetes, there are no adverse cardiac structural changes or cardiac dysfunction, which leads to cardiac hypertrophy only in the presence of comorbid factors such as hypertension [24]. In high-fat diet-induced gestational diabetes mellitus mice, increased ANGPTL8 was accompanied by decreased phosphorylation of IRβ(Tyr1361) and Akt, and silencing ANGPTL8 ameliorated insulin resistance by inhibiting JNK signaling [25]. Furthermore, serum ANGPTL8 was higher in individuals with insulin resistance than in those with insulin sensitivity [26]. These data supported that ANGPTL8 may be a compensatory response factor to balance the effects of pathological stimuli. Some researchers reported that high serum ANGPTL8 was significantly associated with a lower risk of cardiac events and cardiac death in patients with stable CAD [10], but other research showed that high ANGPTL8 levels promoted the development of atherosclerosis [27]. Thus, regarding the action of ANGPTL8 on metabolic-cardiac disease, further work is needed to fully understand its mechanism of action. However, lipid and glucose metabolic disorders did not occur in either Ang II- or TAC-induced cardiac hypertrophy models of WT and ANGPTL8 KO mice in our experiment; therefore, ANGPTL8 may act directly on the heart muscle to inhibit cardiac hypertrophy.

Cardiac fibrosis, as a typical feature of pathological cardiac hypertrophy, is characterized by cardiac collagen accumulation [28, 29]. In contrast to previous studies, ANGPTL8 promoted endothelial barrier dysfunction and proliferation in cancer [21, 30–32]. Our study first demonstrated that ANGPTL8 knockout aggravated cardiac fibrosis and exaggerated fibrotic marker expression in vivo. In contrast, exogenous ANGPTL8 administration or overexpression of ANGPTL8 abolished these changes induced by Ang II in vitro. Previous reports demonstrated that ANGPTL8 KO mice exhibited body weight (BW) and fat mass loss [33], but our results showed that the body weight of ANGPTL8 KO mice was not significantly different from that of WT mice. The potential reason was different week-age mice used. Wang Yan *et al* reported that BW was apparently decreased at the age of 18 weeks, but only a small decrease and no statistical significance was observed in 12-week-old ANGPTL8 KO mice compared with WT mice [34]. In our study, 8-week-old mice were chosen to begin the experiment for a duration of 4 weeks. Furthermore, in line with other reports, 4 weeks of Ang II infusion did not affect body weight or liver weight [24]. Therefore, ANGPTL8 exerted a novel antihypertrophic effect by reducing cardiac fibrosis.

As ANGPTL8 is a secretary protein, whether extracellular or intracellular effects occur is of interest. We found that Ang II directly stimulated ANGPTL8 secretion from both hepatocytes and Kupffer cells, especially hepatocytes, but not from other tissues. Furthermore, the secretion pattern of ANGPTL8 was different in the livers of TAC- and Ang II-treated WT mice. The reason may be that hepatocytes around the central vein of the liver first responded to venous blood pressure, resulting in increased ANGPTL8 expression with radial distribution in TAC. However, Ang II infusion uniformly interacted with receptors on the membrane of hepatocytes, which stimulated even secretion of ANGPTL8. The culture medium supernatant of primary hepatocytes stimulated by Ang II inhibited NRCM size enlargement and hypertrophic markers (ANF, BNP); in contrast, the culture medium supernatant from hepatocytes of ANGPTL8 KO mice had no apparent effects on hypertrophy of NRCMs induced by Ang II. These results demonstrated that ANGPTL8 was mainly derived from hepatocytes and participated in the protection from hypertrophic myocardium induced by Ang II or TAC.

Accumulated evidence has shown that gradually increasing Akt/GSK3β levels could play a crucial role in the process of cardiac hypertrophy from compensatory to decompensatory [35, 36]. Herein, although ANGPTL8 under physiological conditions had no influence on either baseline Akt/GSK3β activation or cardiac structure and function, in the Ang II- or TAC-induced pathological cardiac hypertrophy model, increased p-Akt levels in the heart were observed and further increased following ANGPTL8 knockout in mice, leading to the deterioration of pathological cardiac hypertrophy. Meanwhile, the addition of exogenous ANGPTL8 decreased the activation of Akt and GSK3β in hypertrophic cardiomyocytes induced by Ang II, which could be abrogated by an Akt agonist. ANGPTL8 exerts a cardioprotective effect on pathological cardiac hypertrophy by negatively regulating the Akt/GSK3β signaling pathway.

ANGPTL8 receptors have not been identified to date. Previous studies showed that human immune inhibitory LILRB2 and its mouse ortholog PIRs partially mediated the effect of ANGPTLs on the food-driven hepatic circadian clock, adipogenesis and intracellular liposis [37]. The genes encoding the six PIRAs and the PIRB protein are located on close sites on mouse chromosome 7. Some research has indicated that PIRA and PIRB play different functions in different organs; for example, PIRA blockade or deficiency can block innate myeloid cell memory and attenuate kidney and heart allograft rejection [38], and PIRB promotes leukemia cell differentiation and supports leukemia development [39]. Herein, we found that PIRA1, PIRA2 and PIRB expression was significantly upregulated in the cardiac hypertrophy model in WT mice compared with ANGPTL8 KO mice. Further research demonstrated that ANGPTL8 could interact with PIRB in addition to binding to PIRA1 and PIRA2. Chen’s study showed that ANGPTL8 stimulated adult cardiac progenitor proliferation by PIRB [40], so we further explored whether the receptors PIRB/LILRB3 were involved in the antihypertrophic effect of ANGPTL8. Our results showed that rANGPTL8 significantly elevated the levels of LILRB3 expression after Ang II administration and that si-LILRB3 and anti-LILRB3 abolished the antihypertrophic effect and Akt/GSK3β inactivation of rANGPTL8. Therefore, we speculate that ANGPTL8 can directly interact with PIRs, especially PIRB, to provide a cardioprotective effect against cardiac hypertrophy. Indeed, additional studies are needed to decipher the exact interaction mechanisms involved in PIR activity by ANGPTL8.

There were several limitations in this study. First, global, instead of cell type specific, ANGPTL8 knockout mice were used in our experiment; thus, cardiomyocyte-specific effects of ANGPTL8 were not fully investigated in vivo. Second, because the metabolic kinetics of rANGPTL8 were unknown, we did not perform rANGPTL8 infusion to rescue cardiac hypertrophy in vivo. Third, although we have demonstrated that ANGPTL8 directly binds to PIRs and inhibits the Akt/GSK3β pathway, the detailed molecular mechanism between ANGPTL8 and PIRs should be further clarified.

In summary, ANGPTL8, which binds to LILRB3, negatively regulates the development of pathological cardiac hypertrophy by inhibiting Akt/GSK3β activity. Targeting ANGPTL8 may be a promising strategy for reversing pathological cardiac hypertrophy and provide synergistic effects in combination with AT1 blockers.

## Supplementary information

supplemental material

supplemental material

Original Western Blots

checklist

## Data Availability

All data generated or analyzed during this study are included in the main text and the supplementary information files.
