# Correction: ANGPTL8 is a negative regulator in pathological cardiac hypertrophy

**DOI:** 10.1038/s41419-025-07731-9

**Published:** 2025-06-06

**Authors:** Lin Hu, Jiarui Wei, Yue Zhang, Ziyuan Wang, Junming Tang, Jian Tang, Yujiu Gao, Xiaoqiao Zhang, Yifan Li, Yantong Liu, Shinan Ma, Xingrong Guo, Qiufang Zhang

**Affiliations:** 1 Department of Pharmacology; Hubei Key Laboratory of Embryonic Stem Cell Research; and Department of Geriatrics & General Medicine of Taihe Hospital, Hubei University of Medicine, Shiyan, 442000 Hubei China; 2 College of Pharmacy, Hubei University of medicine, Shiyan, 442000 Hubei China

Correction to: *Cell Death and Disease* 10.1038/s41419-022-05029-8, published online 18 July 2022

We mistakenly used incorrect duplicated DAPI immunostaining images within two group (sham vs TAC, same DAPI single image in TAC group) in Figure 1F, actually, we only mistakenly placed the DAPI existed in the sham group into the TAC group, merge channels of two group were correct, we also replaced the originally corrected DAPI staining in TAC group. This does not impact the conclusions of our experiment.

In addition to this, we acknowledge an error in figure labeling: the β-MHC bands in Figures 4I and 7G were inadvertently duplicated. Importantly, both experiments were actulally conducted under identical conditions(Ctr, AngII,rANGPTL8 and AngII+ANGPTL8), so the observed trends of β-MHC in both experimental groups are consistent, we have corrected the different bands in Figures 4I, this correction does not impact the conclusions of our experiment and the conclusions remain fully supported by the independent replicates (n = 3) confirmed the observed trends in both experimental groups in our previous submitted western blot at our online version of manuscript.

**Original data (Figure 1F)** 
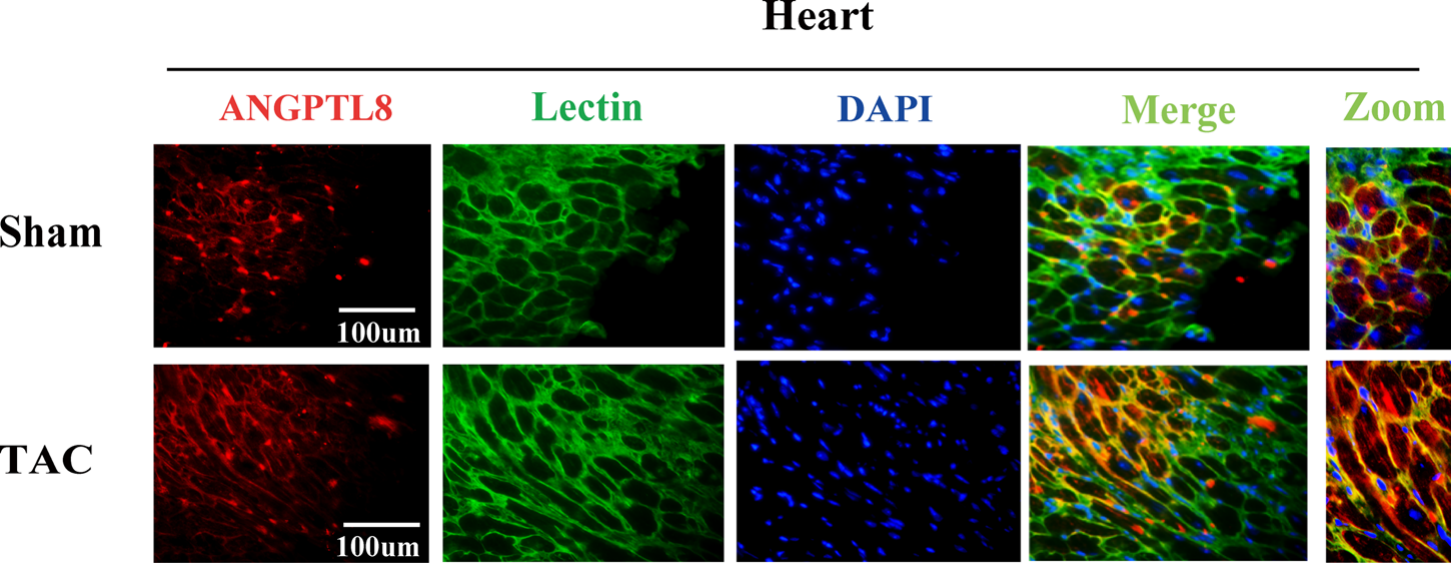


**Amended file_figure 1** 
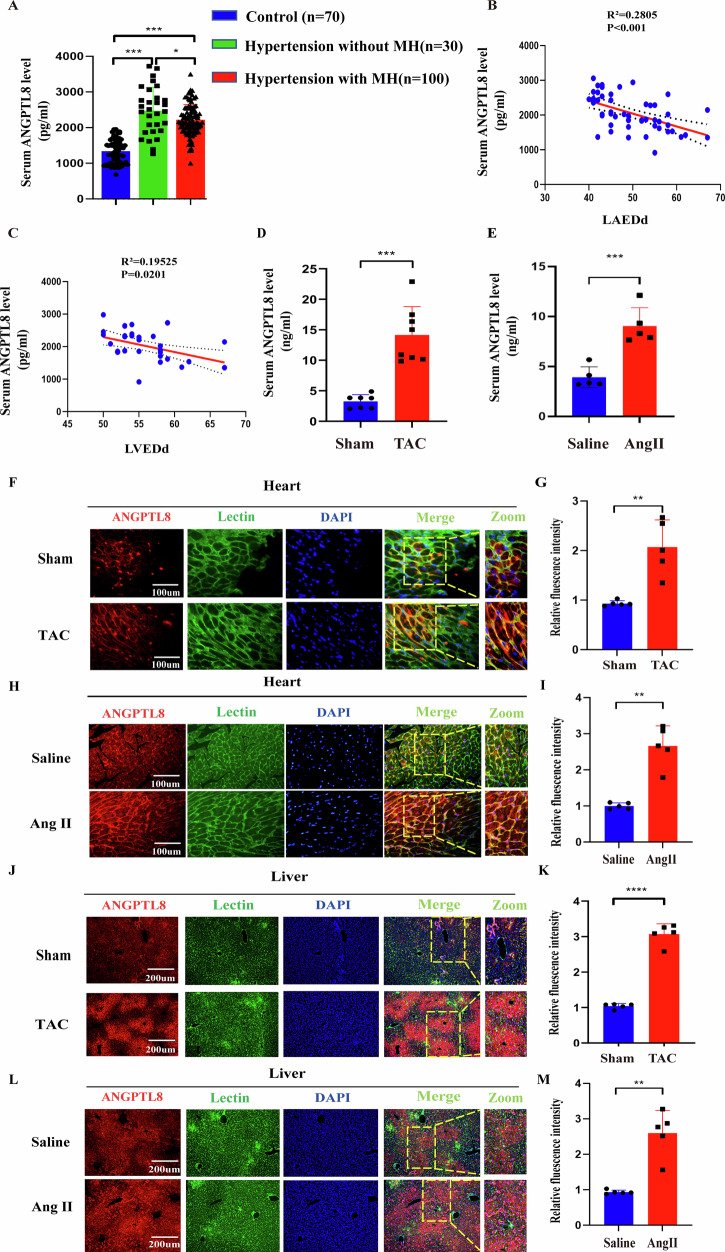


**Original data (Figure 4I)** 
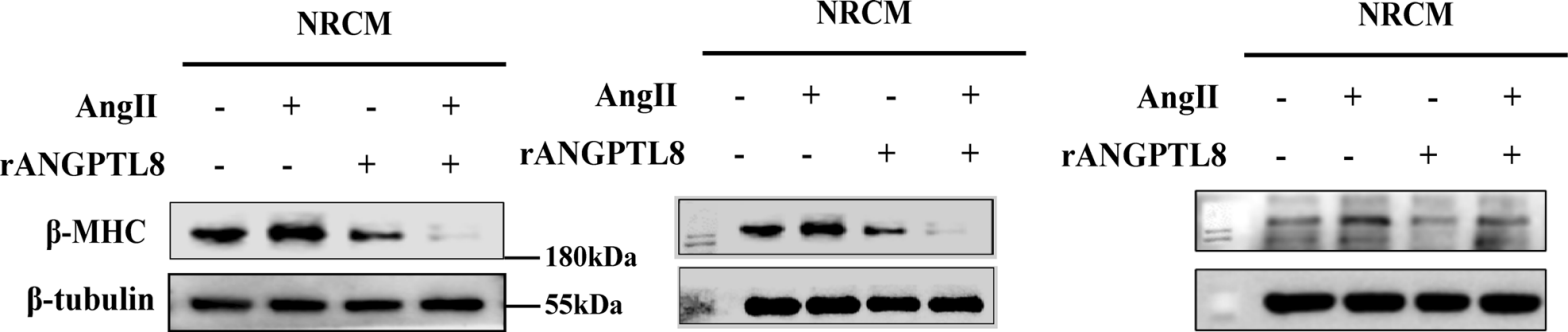


**Amended file-figure 4** 
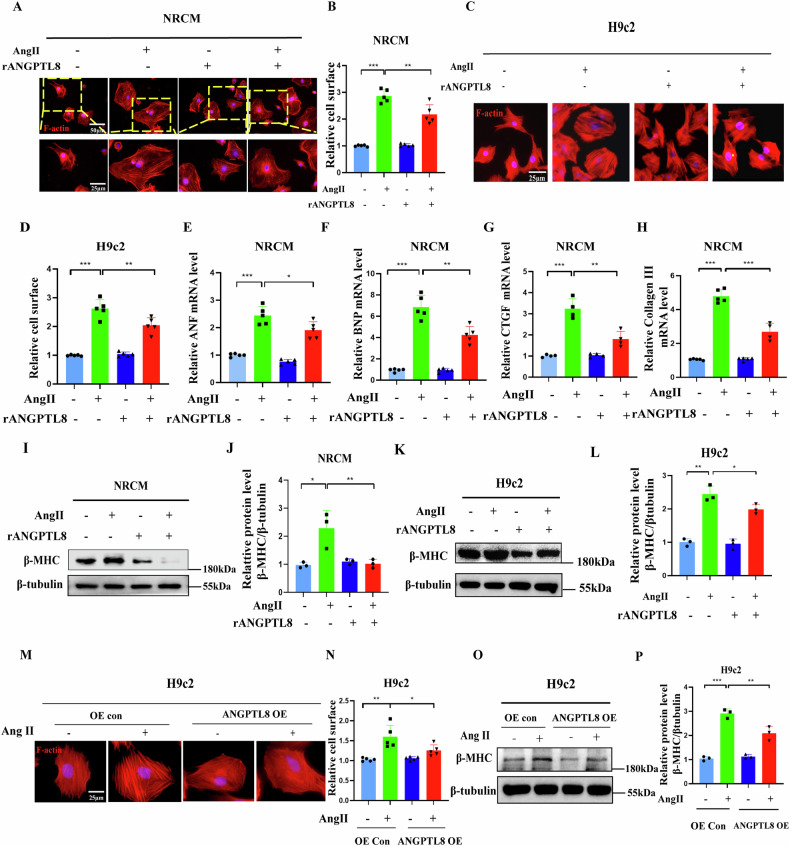


The original article has been corrected.

