# Supplementary material for: ANGPTL8 is a negative regulator in pathological cardiac hypertrophy
Source: Cell Death Dis. 2022 Jul 18;13(7):621. doi: 10.1038/s41419-022-05029-8 (PMC9293964; doi:10.1038/s41419-022-05029-8)
Supplement: Supplementary file 1 — supplemental material [file 41419_2022_5029_MOESM1_ESM.docx]

**Table 1 Comparison of basic physiological parameters in human subjects**

|  | **Control**  **(N=70)** | **Hypertension**  **without MH (N=30)** | **Hypertension**  **with MH (N=100)** |  |
| --- | --- | --- | --- | --- |
| **Median, ages, yrs.** | 52±6.58 | 54±9.53 ^ns^ | 61.45 ± 11.27 ^ns^ |  |
| **Male, %** | 51 | 52 ^ns^ | 54 ^ns^ |  |
| **Examination** |  |  |  |  |
| SBP | 120±10.25 | 155±18.4* | 172 ± 22.4^#^ |  |
| DBP | 65±8.56 | 79.33 ± 4.08* | 80.75 ± 13.23^ns^ |  |
| HR | 70±5.6 | 75.17 ± 8.83* | 77 ± 18.2 ^ns^ |  |
| LVEF (%) | 58±5.65 | 59.35±3.32 ^ns^ | 58.96 ± 8.96 ^ns^ |  |
| LVEDd | 42±3.21 | 41±5.36 ^ns^ | 45±3.52^#^ |  |
| **Laboratory results** |  |  |  |  |
| AST, IU/L | 20±1.25 | 21±3.71 | 33.31±3.27^##^ |  |
| CK, IU/L | 95±10.35 | 103±9.65* | 124±18.94^#^ |  |
| CK-MB, IU/L | 13±3.65 | 12.65±4.25 ^ns^ | 13.46±2.19 ^ns^ |  |
| HBDH, IU/L | 127±8.56 | 118.5±12.74 ^ns^ | 190±33.2^##^ |  |
| LDH, IU/L | 170 ± 6.58 | 168.8 ± 19.35 ^ns^ | 215.9±27.81* |  |
| ACE, IU/L | 78±3.59 | 108.6±5.89 ^ns^ | 62.45±13.45^#^ |  |
| Cysc, IU/L | 0.75 ± 0.115 | 0.739±0.027 ^ns^ | 0.911±0.058^#^ |  |
| **Diseases** |  |  |  |  |
| CAD，% | 0 | 15 | 78 |  |
| MI, % | 0 | 0 | 21 |  |
| Angina, % | 0 | 8 | 69 |  |
| **ANGPTL8(pg/ml)** | 1218±156.74 | 2516±289.21^***^ | 2108±134.86^#^ |  |

All data are presented as the mean ± standard deviation. Abbreviations: SBP, systolic blood pressure; DBP, diastolic blood pressure; HR, heart rate; LVEF, left ventricular ejection fraction; LVEDd, left ventricular end diastolic diameter; AST, Aspartate Transaminase; CK, creatine kinase; CKMB, creatine kinase-MB; HBDH, Hydroxybutyrate Dehydrogenase; LDH, lactic dehydrogenase; ACE, Angiotensin Converting Enzyme; Cysc, cystatin c; CAD, coronary artery disease; MI, Myocardial Infarction; **P*<0.05, ***P*<0.01, compared to control; ^#^*P* <0.05, ^##^*P*<0.01, compared to Hypertension without MH; ns=no significance;

**Supplementary Table 1:** Comparison of basic physiological parameters in hypertensive patients with myocardial hypertrophy(n=100), hypertensive patients without (n=30) and healthy people (control group=Control, n=70)

| **Table 2 Biometric and echocardiographic measurements in experimental mice** | | | | |
| --- | --- | --- | --- | --- |
| **Model 1** | **Continuous Ang II Pump Infusion** | | | |
|  | **Saline** | | **AngII** | |
|  | **WT** | **ANGPTL8 KO** | **WT** | **ANGPTL8 KO** |
| **EF%** | 58.53±1.694 | 56.06±1.286^ns^ | 50.23±3.533* | 41.54±2.747^#^ |
| **FS%** | 34.87±2.899 | 34.46±1.851^ns^ | 24.66±4.036** | 17.74±6.338^###^ |
| **LVIDd, mm** | 2.135±0.0905 | 2.161±0.045^ns^ | 3.563±0.1202** | 3.908±0.2086^#^ |
| **IVSD, mm** | 0.902±0.019 | 0.9874±0.017^ns^ | 1.079±0.2154 | 1.068±0.107^ns^ |
| **LVAWd, mm** | 0.9663±0.466 | 0.953±0.0247^ns^ | 0.998±0.1933 | 1.138±0.1595^ns^ |
| **LVPWd, mm** | 0.9085±0.005 | 0.912±0.051^ns^ | 0.933±0.049* | 1.2402±0.1382^#^ |
| **HW/BW, mg/g** | 4.102±0.125 | 4.136±0.182^ns^ | 5.682±0.3627** | 6.532±1.096^##^ |
| **HW/TL, mg/mm** | 4.890±0.113 | 4.913±0.091^ns^ | 6.463±0.278** | 6.926±0.1801^#^ |

Transthoracic echocardiography was performed on mice at the end of the animal study. EF (ejection fraction) %, FS (fractional shortening) %; LVIDd, diastole left ventricle internal dimension; LVAWd, left ventricular diastole Left ventricular thickness; LVPWd, left ventricular diastole Left ventricular thickness; IVSD, interventricular septal thickness at diastole; HW/BW, heart weight/body weight; HW/TL, heart weight/tibia length. Data presented as mean ± SD.( n =6, AngII group of WT mice *vs* Saline group of WT, **P*<0.05, ***P*<0.01, ****P*<0.001; WT mice *vs* ANGPTL8 KO mice in AngII infusion group, ^#^*P*<0.05, ^##^*P*<0.01, ^###^*P*<0.001, ns= no significant difference).

|  | | | | |
| --- | --- | --- | --- | --- |
| **Model 2** | **Transverse Aortic Constriction** | | | |
|  | **sham** | | **TAC** | |
|  | **WT** | **ANGPTL8 KO** | **WT** | **ANGPTL8 KO** |
| **EF%** | 58.86±1.85 | 57.95±2.23 ^ns^ | 50.67±10.32* | 42±5.37**^#^** |
| **FS%** | 34.40±2.52 | 35.51±2.29 ^ns^ | 27.58±6.834* | 22.55±3.596**^#^** |
| **LVIDd, mm** | 2.065±0.066 | 2.144±0.076 ^ns^ | 2.829±0.906** | 3.459±0.258**^##^** |
| **IVSD, mm** | 0.910±0.009 | 0.914±0.017 ^ns^ | 0.979±0.084 | 0.938±0.087 ^ns^ |
| **LVAWd, mm** | 1.021±0.033 | 1.031±0.045 ^ns^ | 1.240±1.349 | 1.143±0.086^ns^ |
| **LVPWd, mm** | 0.907±0.066 | 0.993±0.43 ^ns^ | 1.349±0.354** | 1.333±0.499 ^ns^ |
| **HW/BW, mg/g** | 4.251±0.250 | 4.293±0.388 ^ns^ | 7.032±0.566* | 8.109±0.214^#^ |
| **HW/BL, mg/mm** | 4.603±0.43 | 4.677±0.193 ^ns^ | 6.868±0.271* | 7.243±0.327^#^ |
| **AD, mm** | 1.279±0.037 | 1.265±0.032 ^ns^ | 1.505±0.089** | 1.707±0.280**^#^** |
| **CO, μl/min** | 41.08±1.692 | 40.36±2.241 ^ns^ | 29.24±8.46** | 25.48±5.329**^##^** |

Transthoracic echocardiography was performed on mice at the end of the animal study. EF ejection fraction %, FS, fractional shortening %; LVIDd, left ventricular diastole left ventricle internal dimension; LVAWd, left ventricular diastole Left ventricular thickness; LVPWd, left ventricular diastole Left ventricular thickness; IVSD, interventricular septal thickness at diastole; HW/BW, heart weight/body weight; HW/TL, heart weight/tibia length. AD, Aortic Diameter; CO, Cardiac Output. Data presented as mean ± SD.( n =10, TAC group of WT mice *vs* Sham group of WT, **P*<0.05, ***P*<0.01, ****P*<0.001; WT mice *vs* ANGPTL8 KO mice in TAC group, ^#^*P*<0.05, ^##^*P*<0.01, ^###^*P*<0.001, ns= no significant difference).

**Supplementary Table 2: Biometric and echocardiographic measurements in experimental mice**

**Table 3 The primer of qPCR used in experiment**

| **Gene** | **Forward 5'-3'** | **Reverse 3'-5'** |
| --- | --- | --- |
| **Rat-LILRB3** | **GGGTCCTCACATGCCAAAGA** | **TCTGGAGGTTCCCTGTCACC** |
| **Rat-ANF** | **AGCGAGCAGACCGATGAA** | **CTCCAATCCTGTCAATCCTACC** |
| **Rat-BNP** | **GCCAGTCTCCAGAACAATCC** | **GCTTGAACTATGTGCCATCTTG** |
| **Rat-β-MHC** | **TGCTGGCACCGTGGACTA** | **GCTTGAGGGAGGACTTCTGG** |
| **Rat-CTGF** | **ACCATGATCCGAGCCAACTG** | **CCGGATGCACTTTTTGCCTT** |
| **Rat-Collagen Ⅲ** | **ATAGCCACCCATTCCTCCG** | **CACCTGCTCCTGTCATTCC** |
| **M-ANF** | **GAGAAGATGCCGGTAGAAGA** | **AAGCACTGCCGTCTCTCAGA** |
| **M-BNP** | **GAGTCCTTCGGTCTCAAGGC** | **ACTTCAGTGCGTTACAGCCC** |
| **M-β-actin** | **GCTGTCCCTGTATGCCTCT** | **TTGATGTCACGCACGATTT** |
| **M-PIRA1** | **AGACTGACACTGGCTTCT** | **GTTAGGCTTCACTGAGAGG** |
| **M-PIRA2** | **ACTACTGGACACCCAGCCTT** | **TGAACCTGTCATAGCTCGGC** |
| **M-PIRB** | **AGACTGACACTGGCTTCT** | **GTTAGGCTTCACTGAGAGG** |
| **M-ANGPTL8** | **TCCTGGGGACAGAAGTCAG** | **AGCTCGAAGGTGTAAAGCG** |
| **H-ANGPTL8** | **GCCTGTTGGAGACTCAGATGGA** | **CGCTGTCCCGTAGCACCTTC** |
| **H-β-actin** | **GGATTCCTATGTGGGCGACGA** | **GCGTACAGGGATAGCACAGC** |

BNP, brain natriuretic peptide; ANF, atrial natriuretic factor; β-MHC, β-myosin heavy chain; CTGF, Connective tissue growth factor; Collagen III, Collagen type III; PIRA1, paired immunoglobulin like receptor A1; PIRA2, paired immunoglobulin like receptor A2; PIRB, paired immunoglobulin like receptor B; ANGPTL8, angiopoietin like protein 8; LILRB3, leukocyte immunoglobulin-like receptor, subfamily B3.

**Supplementary Table 3: The primer of qPCR used in experiment**
