# Supplementary material for: ANGPTL8 is a negative regulator in pathological cardiac hypertrophy
Source: Cell Death Dis. 2022 Jul 18;13(7):621. doi: 10.1038/s41419-022-05029-8 (PMC9293964; doi:10.1038/s41419-022-05029-8)
Supplement: Supplementary file 2 — supplemental material [file 41419_2022_5029_MOESM2_ESM.docx]

Supplementary Information for

**ANGPTL8 is a negative regulator in pathological cardiac hypertrophy**

Lin Hu^1^, Jiarui Wei^1^, Yue Zhang^1^, Ziyuan Wang^2^, Junming Tang^1^, Jian Tang^1^, Yujiu Gao^1^, Xiaoqiao Zhang^1^, Yifan Li^1^, Yantong Liu^1^, Shinan Ma^1^, Xingrong Guo^1^*, Qiufang Zhang^1^*

**Affiliations**

1 Department of Pharmacology; Hubei Key Laboratory of Embryonic Stem Cell Research; and Department of Geriatrics & General Medicine of Taihe Hospital; Hubei University of Medicine, Shiyan, 442000, Hubei, China

2 College of Pharmacy, Hubei University of medicine, Shiyan, 442000, Hubei, China

*Corresponding author: Qiufang Zhang, Ph.D. Email: zqf1112000@163.com 30 Renmin South Road, Shiyan, Hubei, China, 442000; Xingrong Guo, Ph.D. E-mail: gxrdl@hbmu.edu.cn (X. Guo).

**This PDF file includes:**

Supplementary Fig. 1 to 7

Supplementary Experimental methods 1 to 4

**
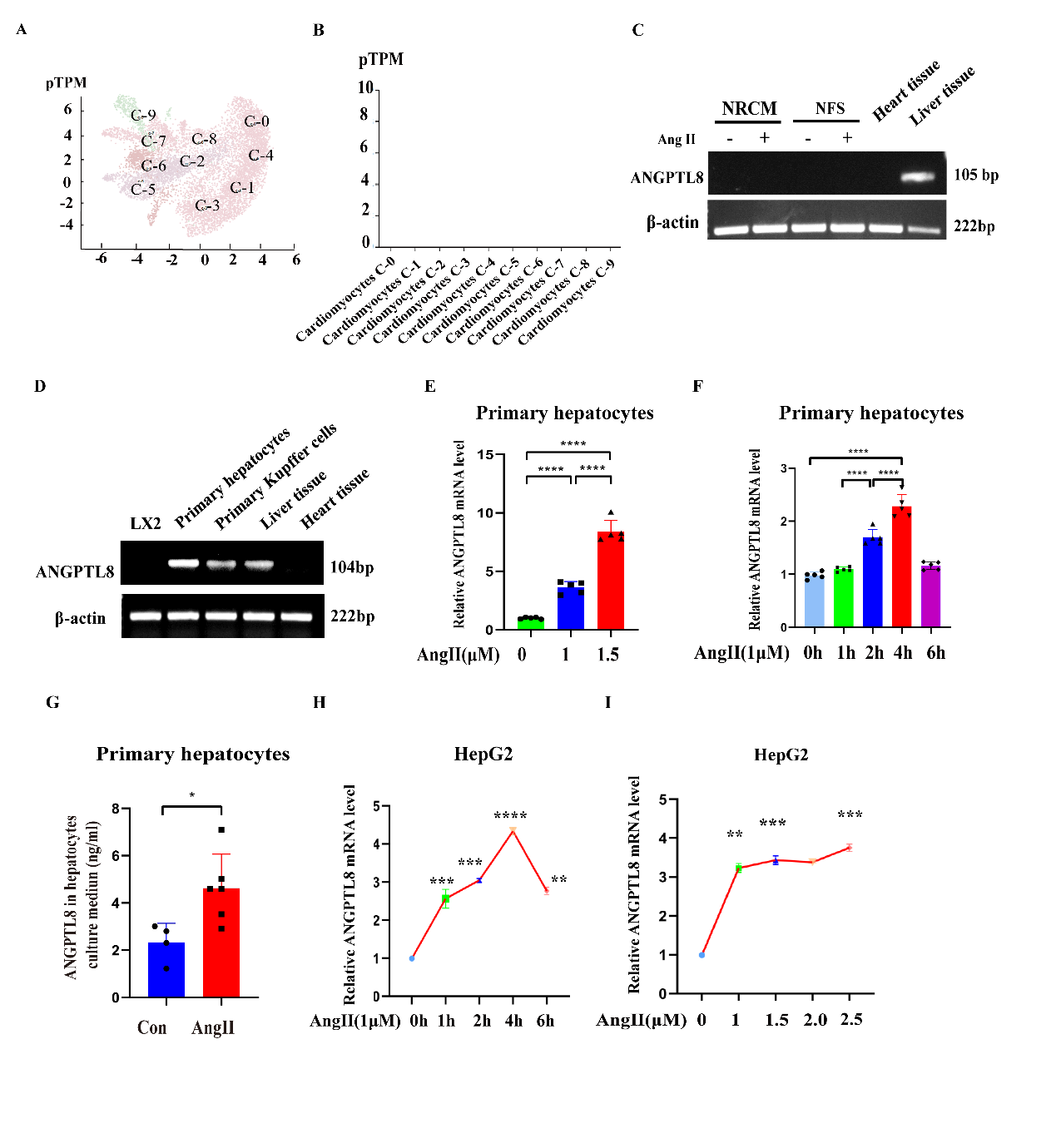
**

**Supplementary Figure. 1: ANGPTL8 is secreted from primary hepatocytes. (A-B)**. ANGPTL8 expression in different heart areas by single myocyte sequences; data were obtained from the Human Single Cell Sequencing Database. **(C)**. The expression of ANGPTL8 mRNA in NRCM and neonatal rat cardiac fibroblasts (NFS) treated with AngII and in heart and liver tissue was detected by RT–PCR. **(D)**. The expression of ANGPTL8 mRNA in liver-related cells, such as primary hepatocytes, primary Kupffer cells and LX2 cells. **(E-F)** The mRNA level of ANGPTL8 was upregulated in primary hepatocytes induced by Ang II in a time- and concentration-dependent manner (n=5). **(G)**. The concentration of ANGPTL8 in the culture medium of primary hepatocytes exposed to AngII (1 µM) for 12 hours was determined by ELISA (n=4~6 mice per group). The mRNA level of ANGPTL8 was upregulated in HepG2 cells induced by AngII in a time- and concentration-dependent manner (n=3). (ns=no significance, ***P<0.01, ***P<0.001，****P<0.0001*)

**
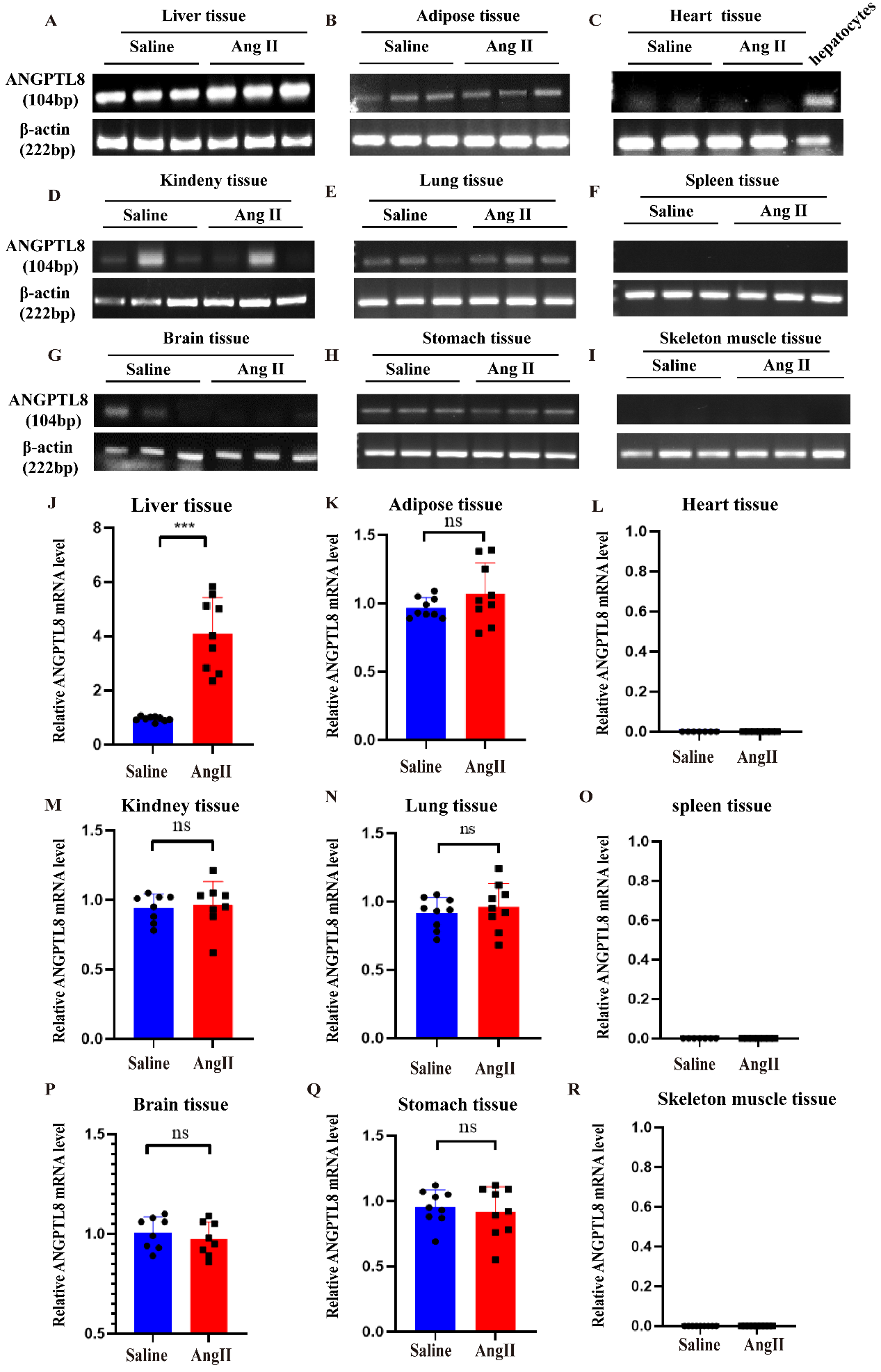
**

**Supplementary Figure. 2: ANGPTL8 mRNA expression in different tissues in WT mice with cardiac hypertrophy induced by AngII.** (A-I). The mRNA expression of ANGPTL8 in different organ tissues in WT mice with cardiac hypertrophy induced by AngII was detected by RT–PCR. Liver tissue (A), adipose tissue (B), heart tissue (C), kidney tissue (D), lung tissue (E), spleen tissue (F), brain tissue (G), stomach tissue (H), skeleton muscle tissue (I). (J-S) The mRNA expression of ANGPTL8 in different organ tissues in WT mice with cardiac hypertrophy induced by AngII was detected by qPCR. liver tissue (J), adipose tissue (K), heart tissue (L), kidney tissue (M), lung tissue (N), spleen tissue (O), brain tissue (P), stomach tissue (Q), skeleton muscle tissue (R); (n=3 mice in each group, three duplicates in each mouse, ns=no significance, ***P<0.01*)

**
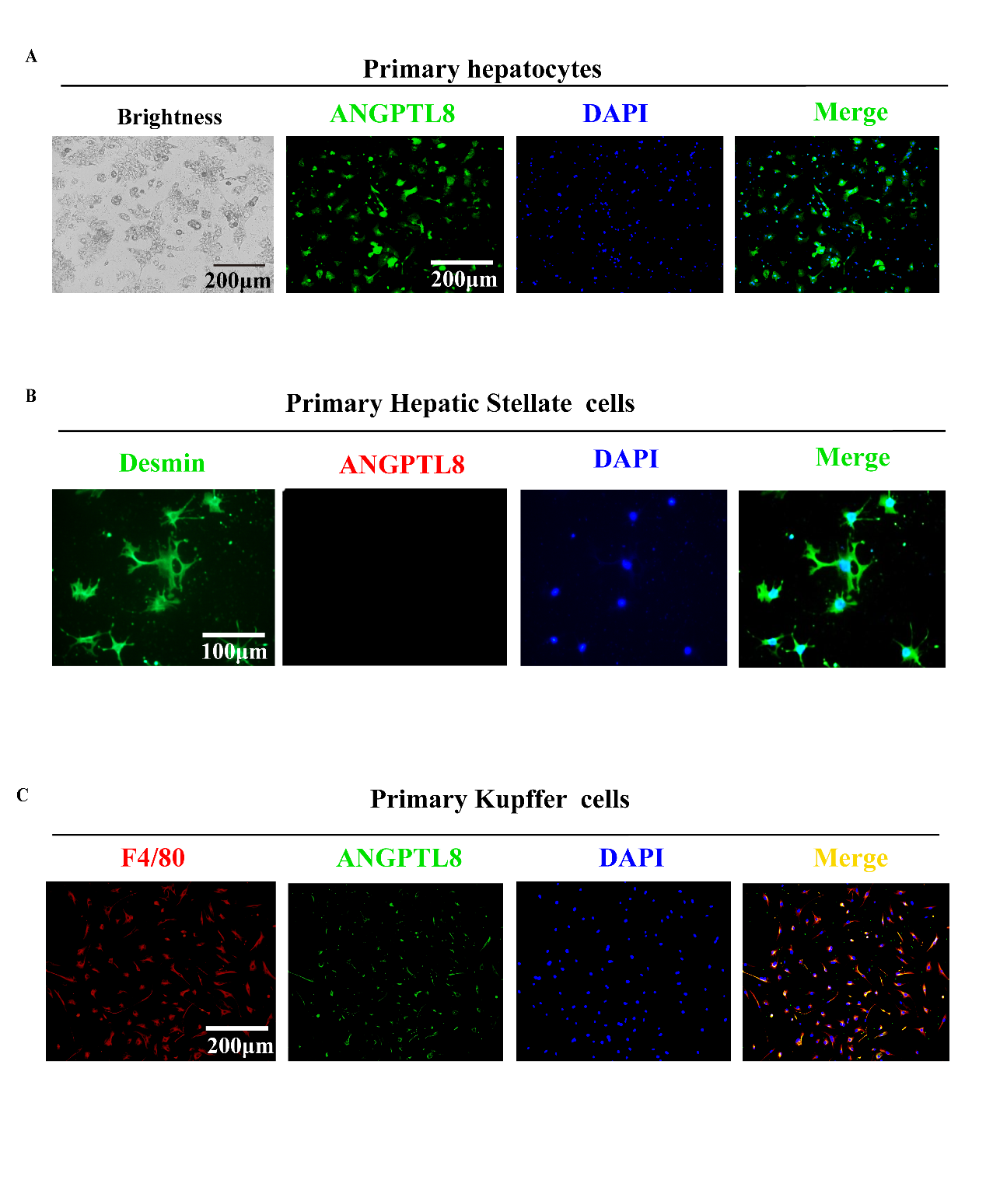
**

**Supplementary Figure. 3: ANGPTL8 expression in primary liver-related cells.** (A). Representative images of immunofluorescence with an anti-ANGPTL8 antibody (nuclei stained by DAPI) in primary hepatocytes isolated from WT mice (scale bar, 200 μm). (B). Representative images of immunofluorescence with anti-ANGPTL8 and desmin antibodies in primary hepatic stellate cells isolated from WT mice (scale bar, 100 μm). (C). Representative images of immunofluorescence with anti-ANGPTL8 and anti-F4/80 antibodies in primary Kupffer cells isolated from WT mice (scale bar, 200 μm).

**
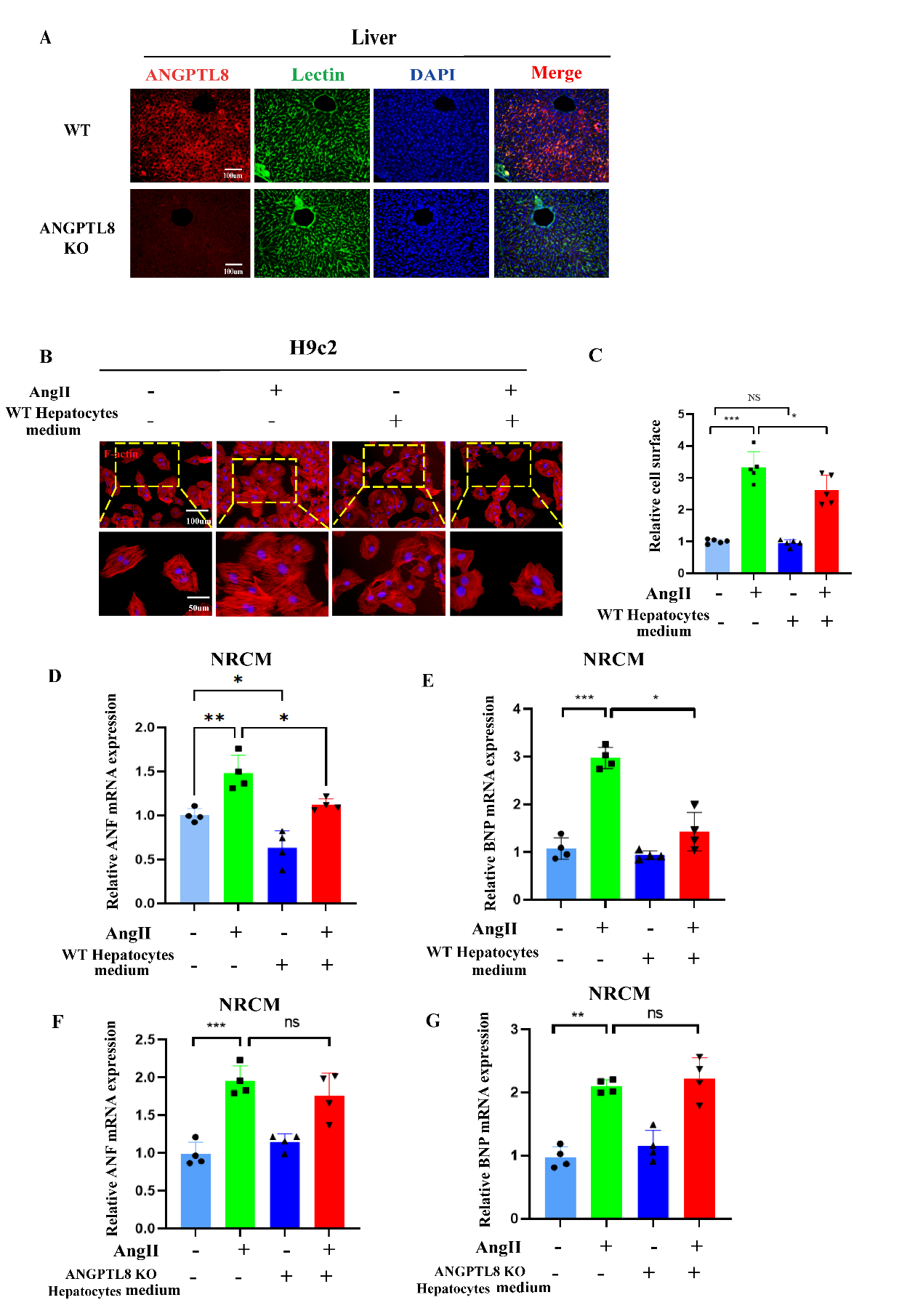
**

**Supplementary Figure 4: ANGPTL8 secreted from hepatocytes attenuated cardiomyocyte hypertrophy induced by AngII.** (A). Representative images of immunohistochemistry with an anti-ANGPTL8 antibody in slices from the indicated liver (n=3, scale bar, 100 μm). (B-F). First, we collected the culture medium of hepatocytes of WT mice after treatment with AngII (1 µM) for 12 h and then used this collected culture medium and AngII(1 µM) to incubate H9c2 or NRCM cells for 48 h. (B-C). Representative images of rhodamine phalloidin staining and quantitative results of the cell surface of H9c2 cells in the indicated groups (n>50 cells in each group, 5 independent experiments, scale bar, 100 μm and 50 μm, respectively). (D-E). qPCR was performed to determine the mRNA levels of ANF (D) and BNP (E) in the indicated groups (n=4 independent experiments). (F-G). Culture medium of primary hepatocytes of ANGPTL8 KO mice treated with AngII (1 µM) for 12 h was collected and then used to incubate NRCM cells with AngII (1 µM) for 48 h. qPCR results of ANF mRNA (F) and BNP mRNA (G) in the indicated group (n=4 independent experiments). NS=no statistical significance **P<0.05, **P<0.01, ***P<0.001.*

**
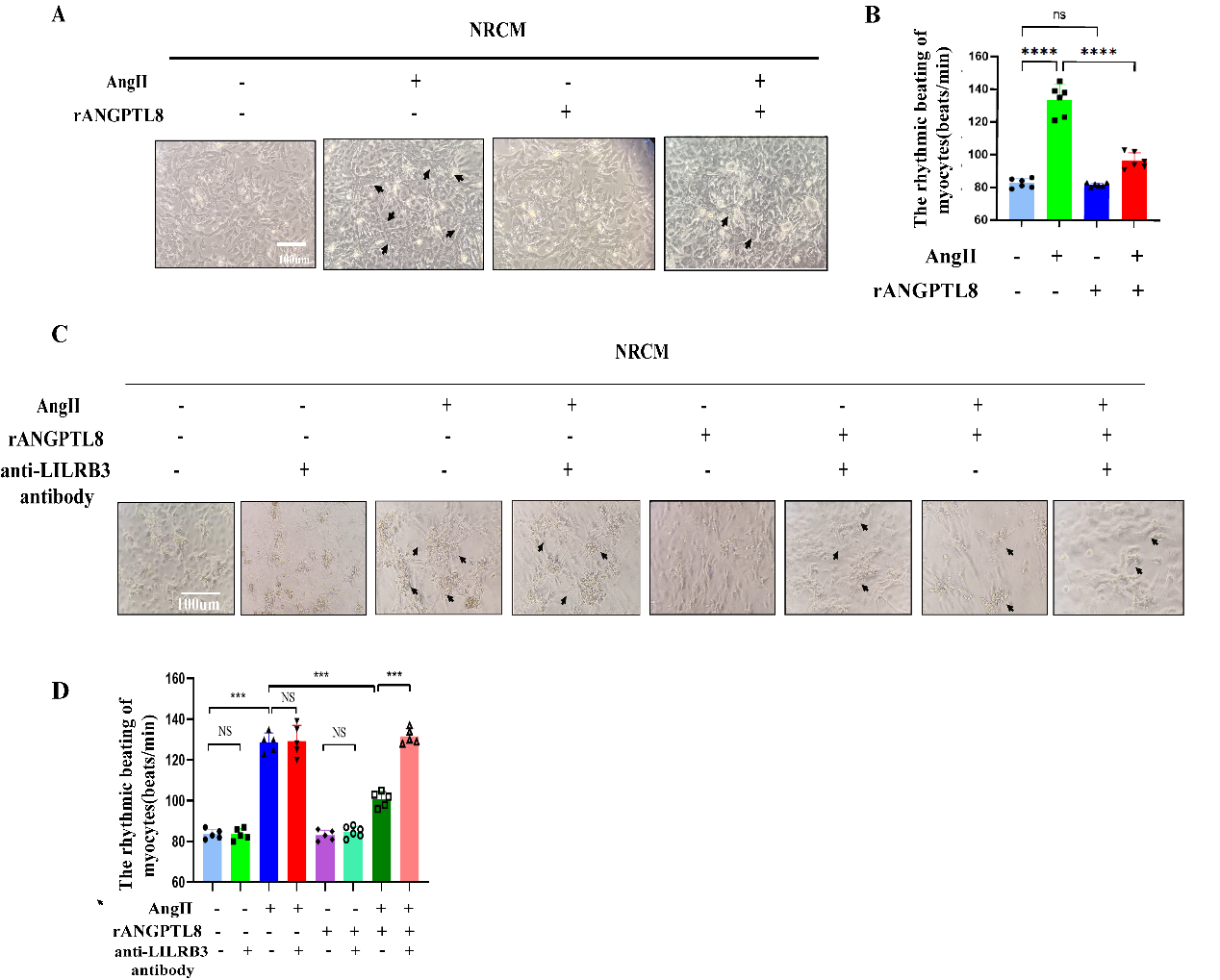
**

**Supplementary Figure. 5: ANGPTL8 ameliorated the rhythmic beats of NRCM and hypertrophic morphology.** (A). Morphological changes in NRCM under Ang II and/or rANGPTL8 treatment (the black arrow points to hypertrophic cells). (B). The statistical results of rhythmic beats of NRCM at 48 h after treatment with Ang II or/and rANGPTL8 (n=6). (C-D) The morphological changes in NRCMs and the beating frequency of NRCMs 48 h after treatment with Ang II and/or rANGPTL8 plus anti-LILRB3 antibody were analyzed in the indicated groups. (Black arrows point to hypertrophic cells) (n=10 fields in each group and 5 independent experiments, scale bar, 100 μm, NS or ns=no statistical significance; ****P<0.001*)

**
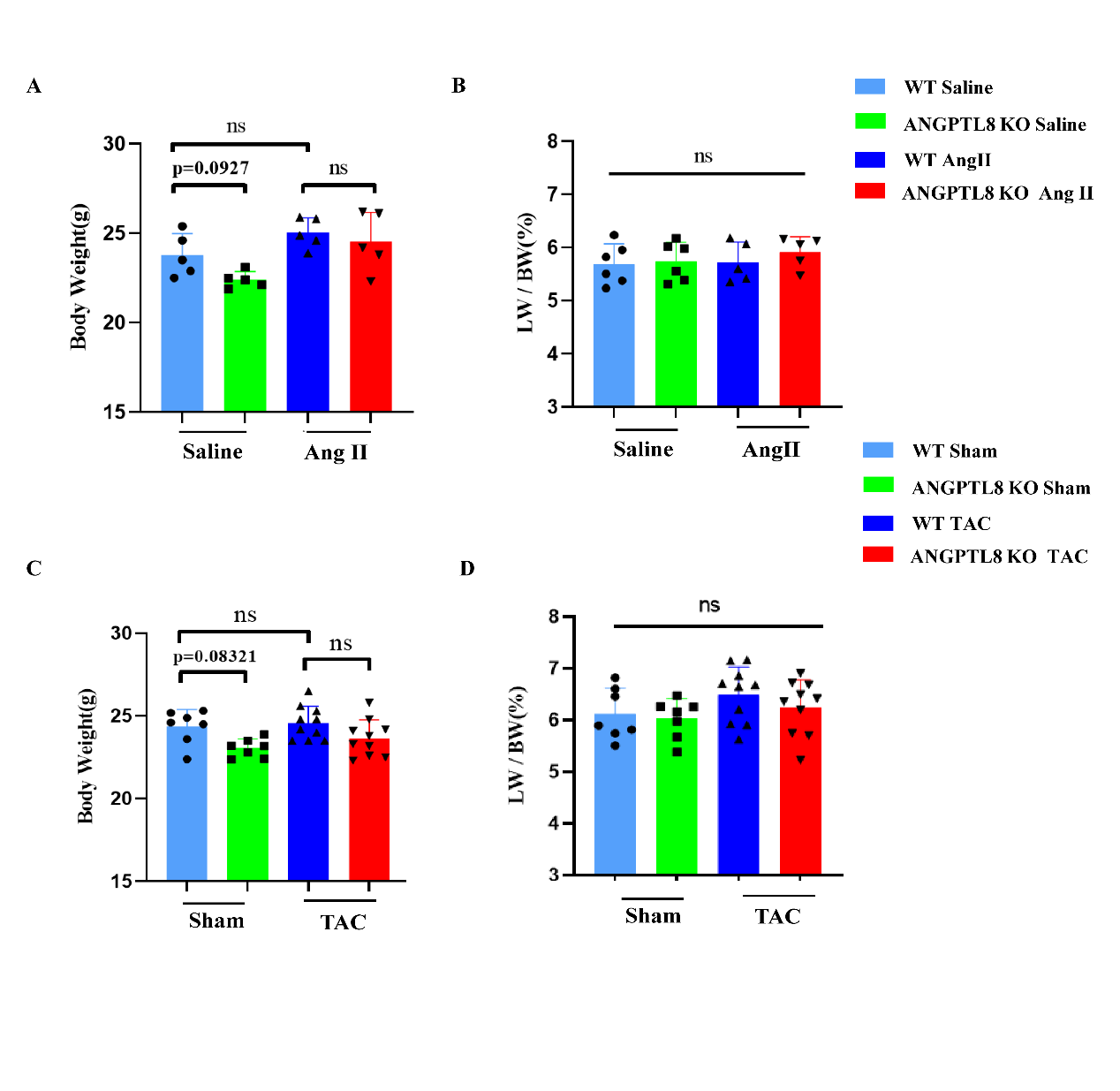
**

**Supplementary Fig. 6: The effects of ANGPTL8 knockout on body weight and liver weight index.** (A) Body weight (BW) and the ratios of (B) liver weight (LW)/BW were analyzed in WT and ANGPTL8 KO mice subjected to AngII or saline infusion for 4 weeks (n=5~6). (C-D). (C) BW and (D) LW/BW were analyzed in WT and ANGPTL8 KO mice subjected to TAC or sham for 4 weeks (n=7~10). (ns=no statistical significance; **P<0.05*)

**
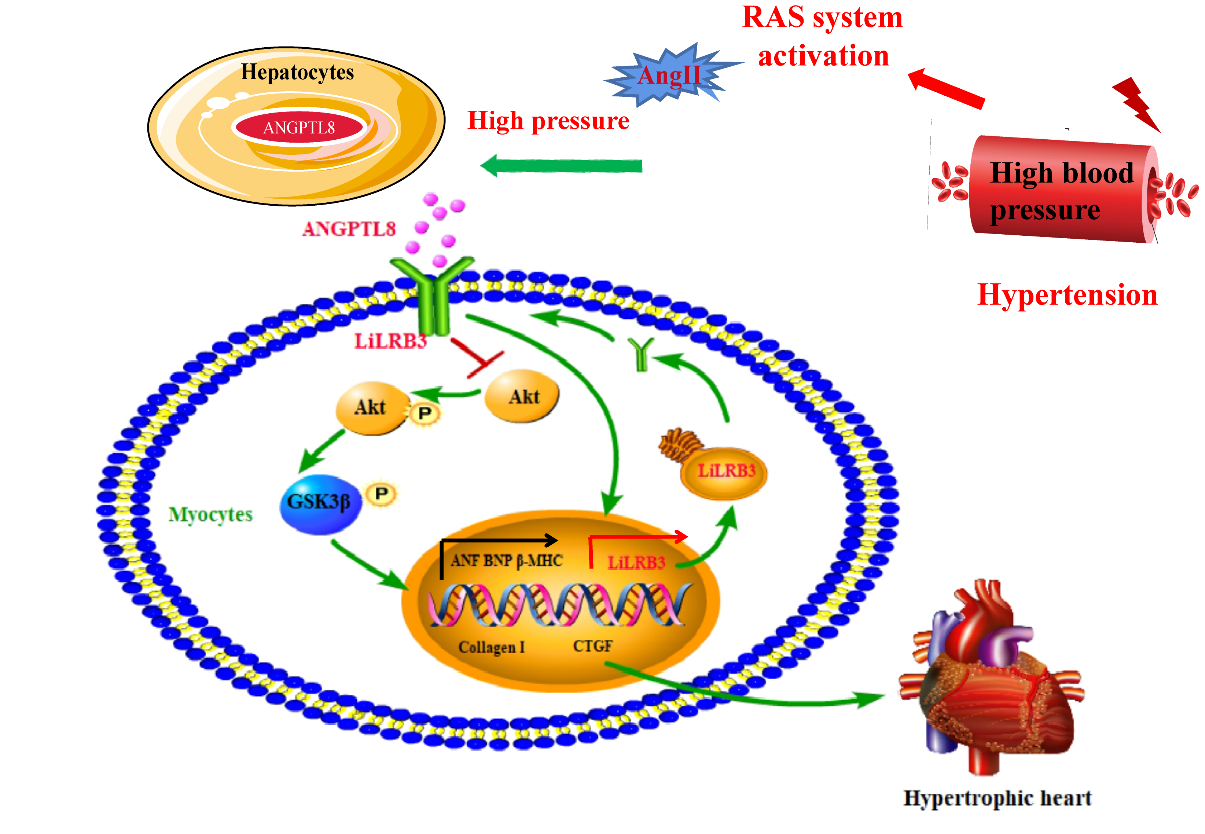
**

**Supplementary Figure. 7:** **Schematic action model of ANGPTL8 attenuating pathological cardiac hypertrophy.**

**Supplementary Experimental methods 1 to 4**

**1. Primary mouse hepatocyte culture**

Primary mouse culture of hepatocyte isolation [1]. In brief, primary hepatocytes were isolated from male C57BL/6 mice (8 weeks old). The mice were anesthetized with 1% pentobarbital sodium (50 mg/kg i.p.). Then, the liver was perfused with the washing anterior perfusion solution (HBSS free with Ca2+ and Mg2+, 0.5 mM EDTA) at a speed of 4-8 ml/min via the portal vein, followed by posterior digestion buffer (collagenase IV, 0.5 mg/ml, Sigma, USA). Approximately 4 min later, the well-digested livers were completely removed from the mice. Digestion from liver tissue was halted by a prepared ice–cold DMEM solution containing 10% FBS followed by centrifugation at 50 g and 4°C for 5 min. The cell pellet was washed with DMEM and centrifuged as described above 2-3 times. Add 10 mL 45% Percoll solution into the cell sedimentation and mix thoroughly by inverting the tube several times. After centrifugation at 25 g for 5 min at 4°C, most of the supernatant was aspirated. Then, the cells were suspended in DMEM high glucose containing 10% FBS and 1% antibiotics and seeded into gelatin-coated plates. The hepatocytes were allowed to adhere in culture plates for 2–4 h at 37°C under a humidified atmosphere with 5% CO2. Then, the nonadherent cells were removed by washing, and the adherent cells were cultured for another 12-24 h for further study.

**2. Primary hepatic Kupffer cell culture**

Primary hepatocytes isolated from male C57BL/6 mice were subjected to the procedures described above for hepatocyte isolation [2]. On the basis of the first centrifugation, we collected the supernatant cell suspension after the first centrifugation and separated primary liver macrophages by Percoll density gradient separation. In brief, the cell pellet was transferred to a clean 50 mL tube and centrifuged at 500 × g for 5 min at 4°C. Then, the supernatant was discarded, and the cell pellets were resuspended in 8 ml of PBS by gently pipetting with 1 ml of a micropipette. The suspension was then carefully layered onto a 25% two-step Percoll gradient (LABLEAD, China) in a 50 mL conical centrifuge tube and mixed thoroughly. Then, the contained 25% Percoll cell suspension was placed into a 50 ml conical centrifuge tube precontaining 3 ml 50% Percoll and mixed thoroughly again. The tube was centrifuged at 1000 × g for 15 min at 4°C. The upper layer (supernatant) was carefully removed from the tube and discarded. The middle layers in the tube were carefully collected into a new 15 mL tube and filled with 5 ml PBS. The middle layers were centrifuged for 5 min at 600 g and 4°C. The supernatant was discarded, and the cell pellets were resuspended. F4/80-positive cells (ab6640, Abcam, USA) were identified as Kupffer cells (KCs) by immunofluorescence analysis to reach approximately 95%.

**3. Primary hepatic stellate cell (HSC) culture**

First, a liver cell suspension was prepared by constant portal vein perfusion as described above. The supernatant cell suspension was centrifuged at a low temperature of 50 g/min for 3 min and then centrifuged at a low temperature of 200 g/min for 8 min. The cell precipitate was taken, and 8 ml serum-free DMEM was added to resuspend and mix evenly. Next, 4 ml of cell suspension was added to 35% and 25% Percoll separation solution in equal proportions and centrifuged at a low temperature of 500 g/min for 10 min. The cell layer was carefully absorbed between 25% and 35% Percoll, and serum-free DMEM was added for culture. The medium was centrifuged at 500 g/min for 5 min, and finally, the supernatant was added to DMEM containing 10% fetal bovine serum for culture. Desmin-positive cells (16520-1-AP, protein, China) were identified as hepatic stellate cells (HSCs) by immunofluorescence analysis to reach approximately 95%.

**4. Construction of the chimeric receptor expression system and Luciferase Reporter Gene Assay**

To explore the direct interaction between ANGPTL8 and PIRs, we cooperated with Huazhong University of Science and Technology to construct a chimeric receptor expression system of the PIR family (PIRA1, PIRA2, PIRA3, PIRA5, PIRA7 and PIRB) [3]. Luciferase was measured using the Firefly Luciferase Reporter Gene Assay Kit (Beyotime Biotechnology, Jiangsu, China, RG051S). For the luciferase assay performed in HEK-293T cells, cells in 96-well plates were cotransfected with 300 ng/well luciferase reporter constructs of PIRs as above, 300 ng/well FLAG-ANGPTL8 plasmid or empty plasmid control using 1.8 μl Lipofectamine 8000 and 60 μl α-MEM medium. Two hundred nanograms/well empty luciferase plasmids served as the internal control. After 48 h of cotransfection, the cells were incubated with luciferin agent for 5 min in equal volumes following the instructions of Bright LumiTM. The fluorescence intensities of different supernatants with luciferin agent were detected by an RF-5301PC fluorophotometer (SHIMADZU, Japan).

**References**

1. Charni-Natan, M and Goldstein I, *Protocol for Primary Mouse Hepatocyte Isolation.* STAR Protos, 2020. **1**(2): p. 100086.

2. Wang, Q, Ou Y, Hu G, Wen C, Yue S, et al. *Naringenin attenuates nonalcoholic fatty liver disease by downregulating the NLRP3/NF-kappa B pathway in mice.* Br J Pharmocol, 2020. **177**(8): p. 1806-1821.

3. Dai H, Lan P, Zhao D, Abou-Daya K, Liu W, Chen W, et al. *PIRs mediate innate myeloid cell memory to nonself MHC molecules*. Science. 2020 Jun 5;368(6495):1122-1127.
