# Supplementary material for: ANGPTL8 is a negative regulator in pathological cardiac hypertrophy
Source: Cell Death Dis. 2022 Jul 18;13(7):621. doi: 10.1038/s41419-022-05029-8 (PMC9293964; doi:10.1038/s41419-022-05029-8)
Supplement: Supplementary file 3 — Original Western Blots [file 41419_2022_5029_MOESM3_ESM.pdf]

Original western Blots in Figure 4:

Repeat 1

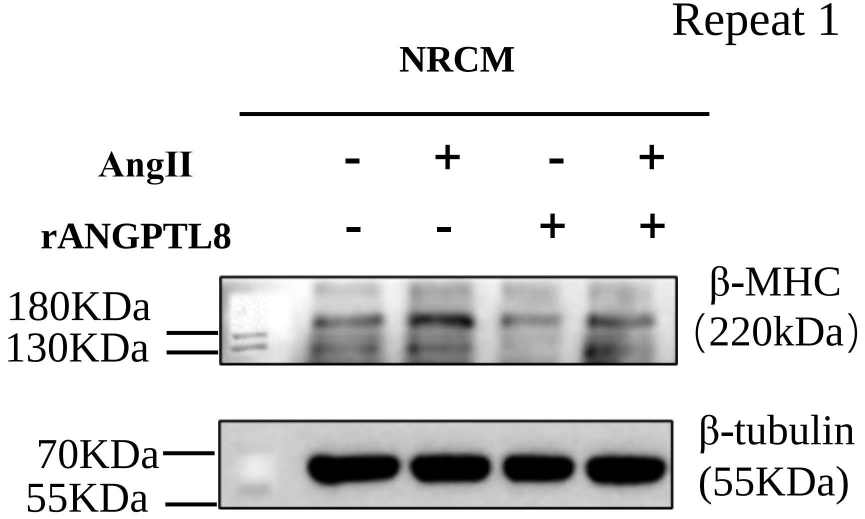

Repeat 2

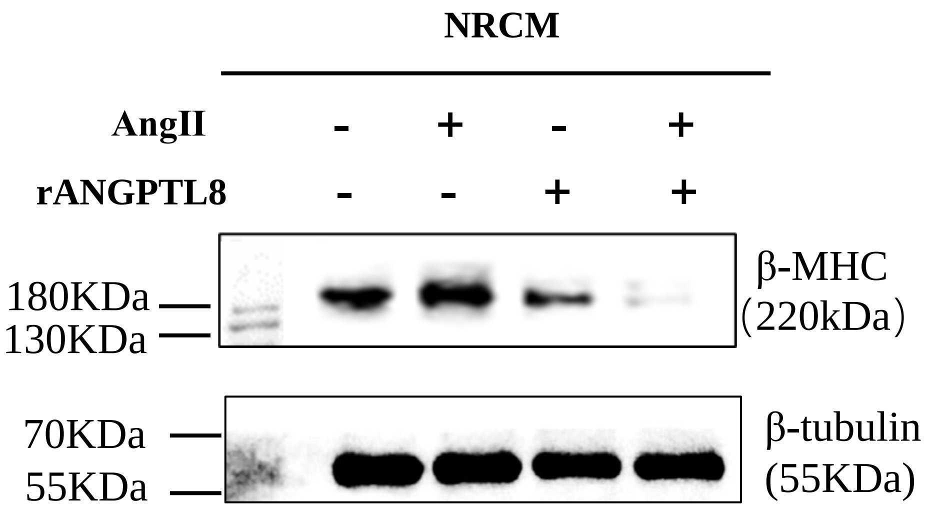

Repeat 3

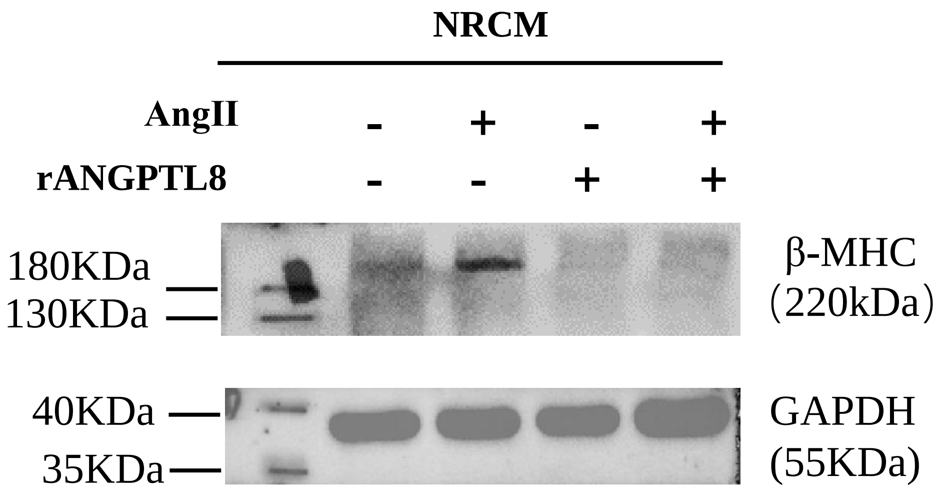

Original western Blots in Figure 4:

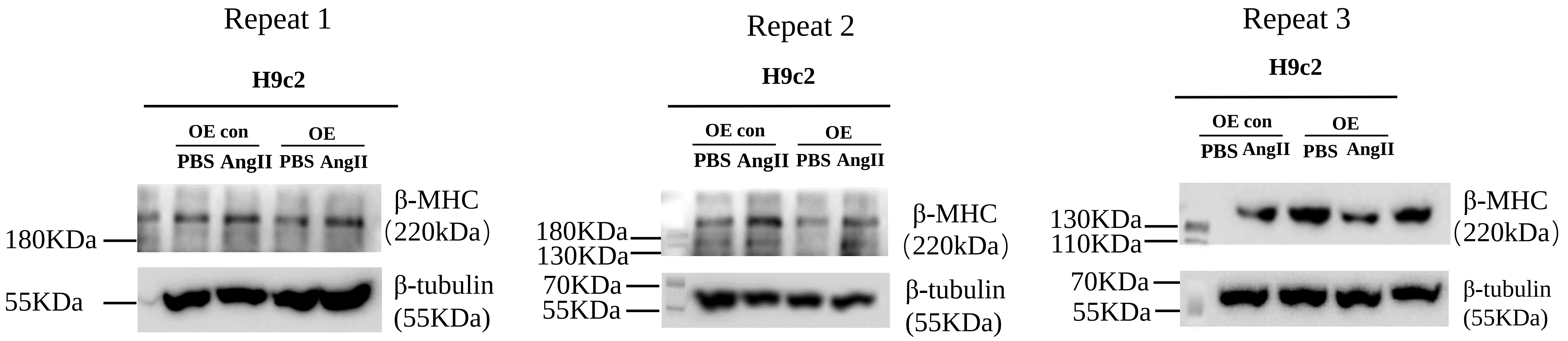

Original western Blots in Figure 4:

Repeat 1

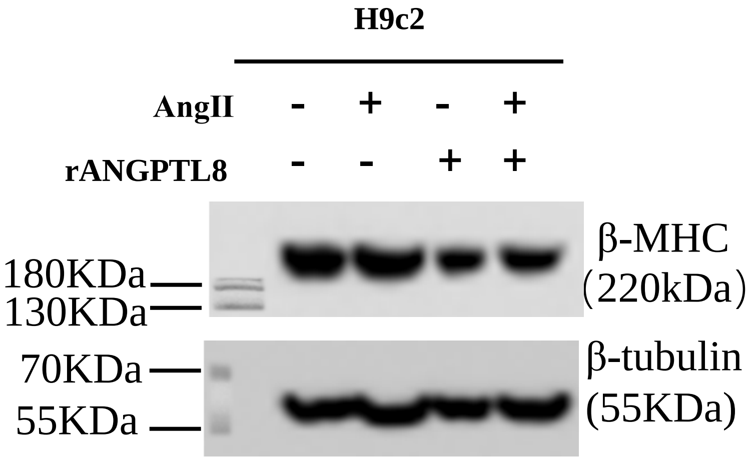

Repeat 2

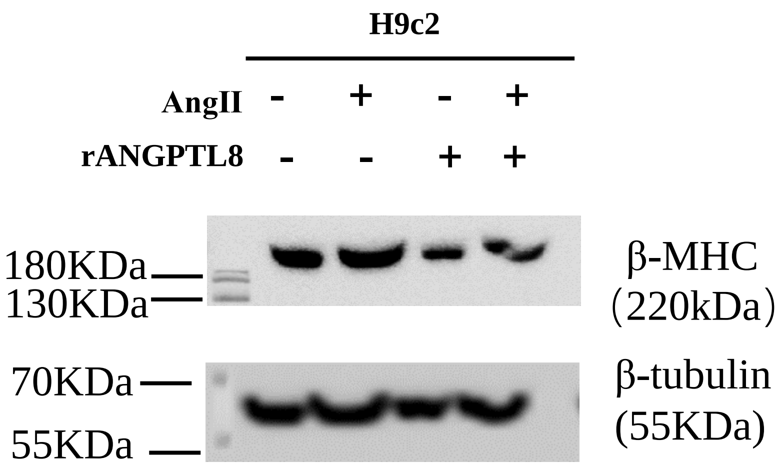

Repeat 3

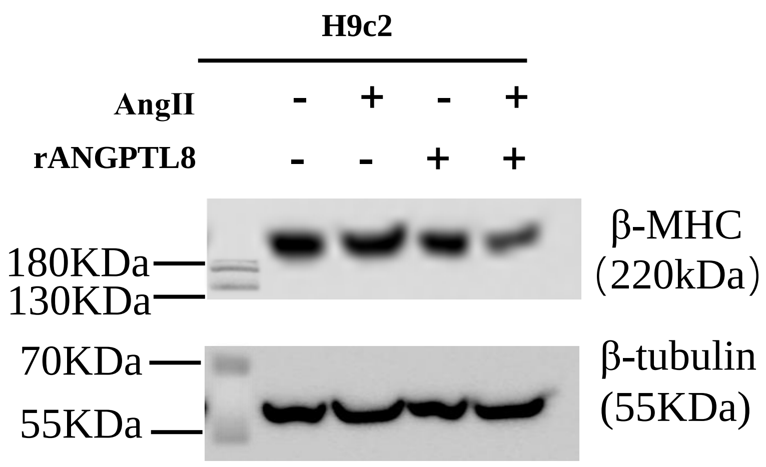

Original western Blots in Figure 4:

Repeat 1

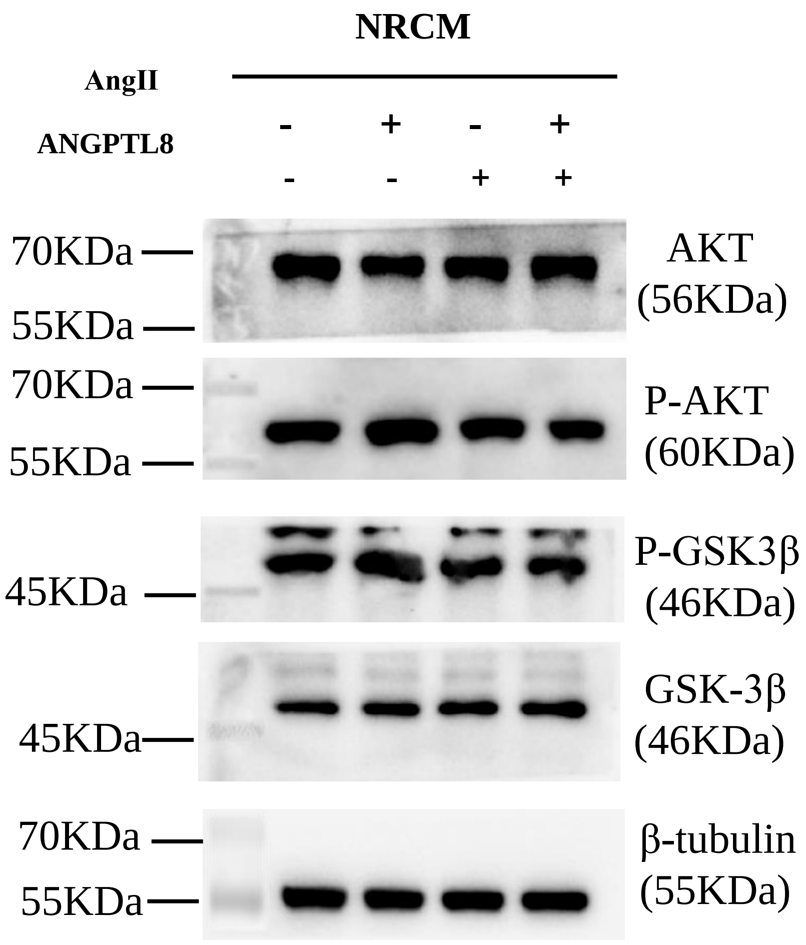

Repeat 2

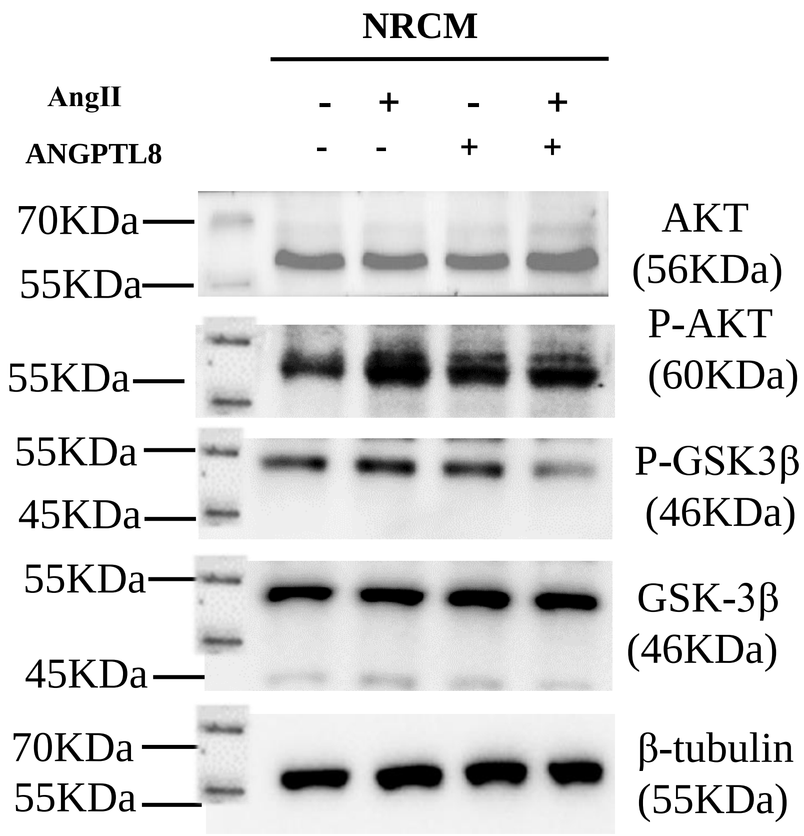

Repeat 3

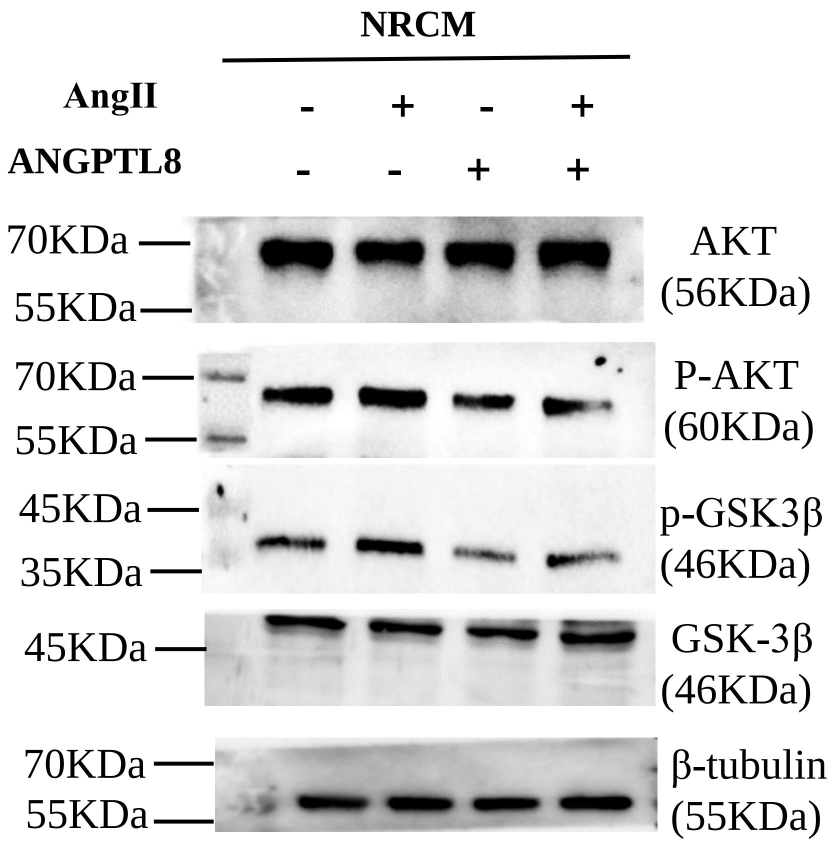

Original western Blots in Figure 3:

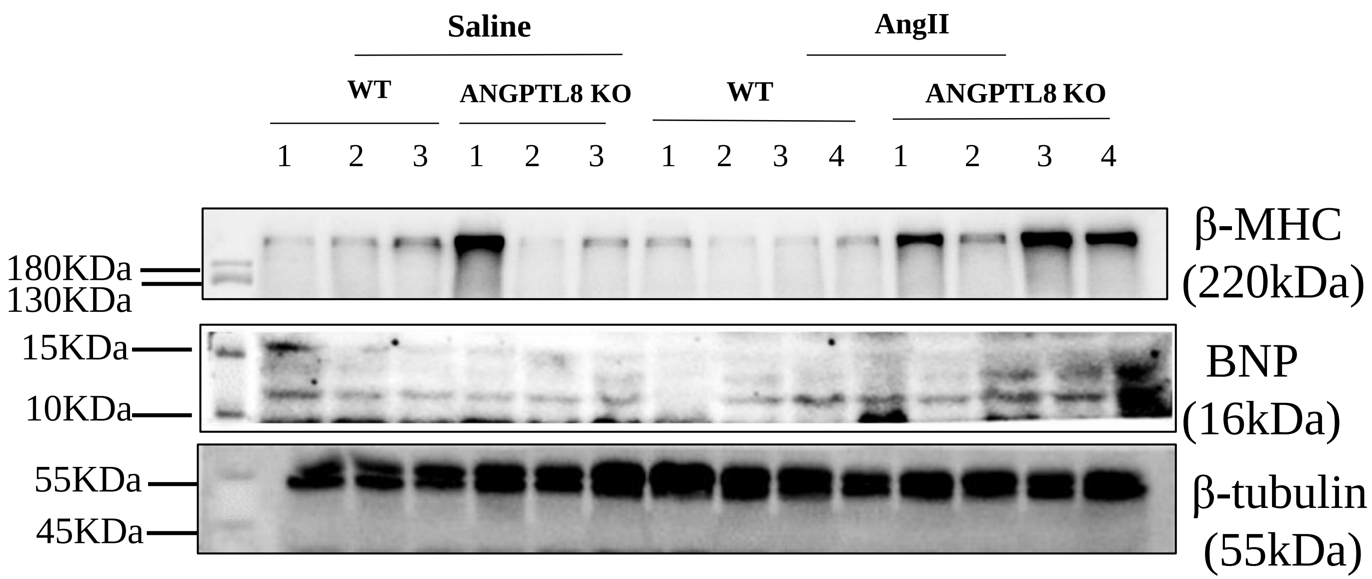

Original western Blots in Figure 6:

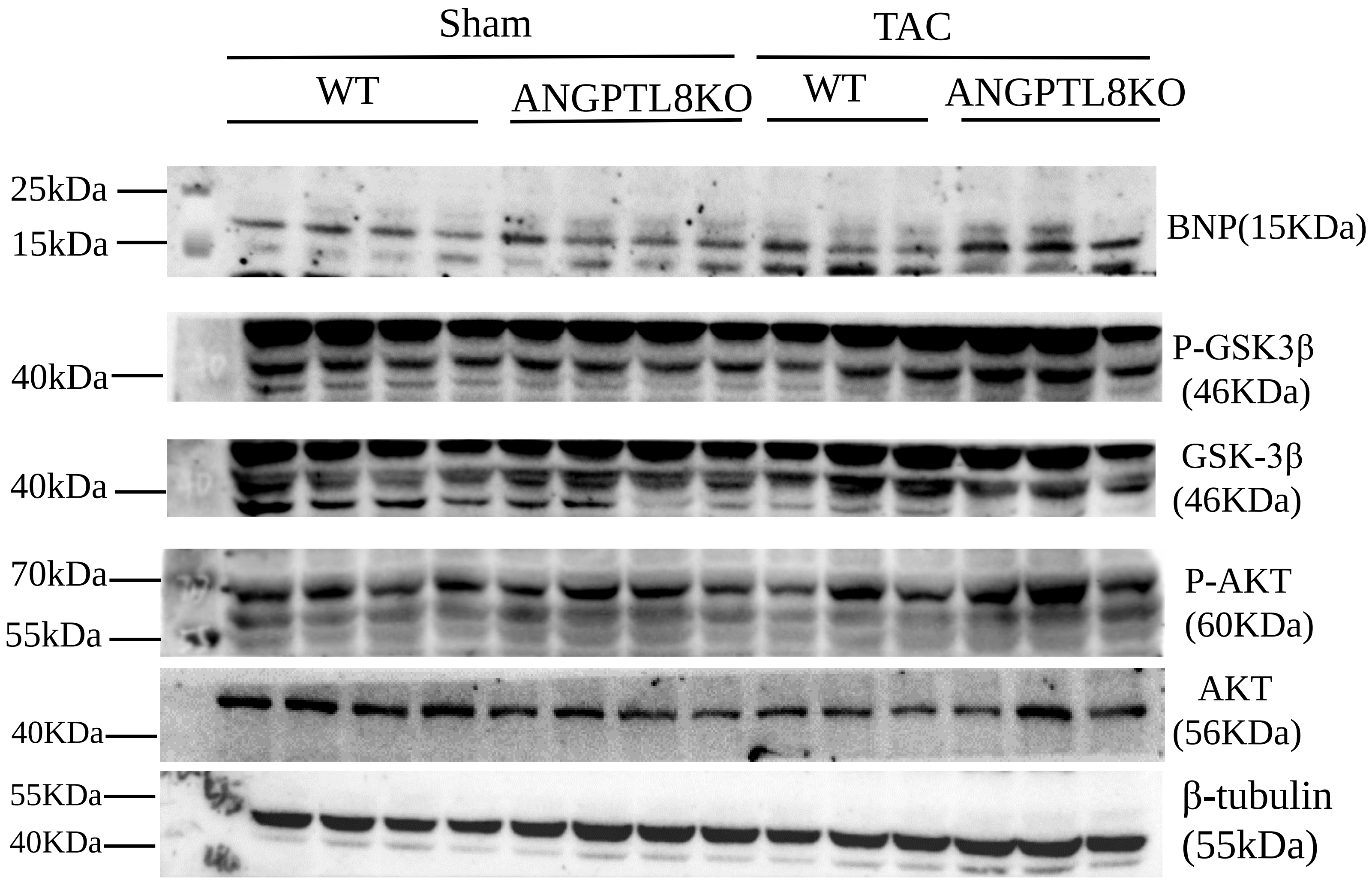

Original western Blots in Figure 7:

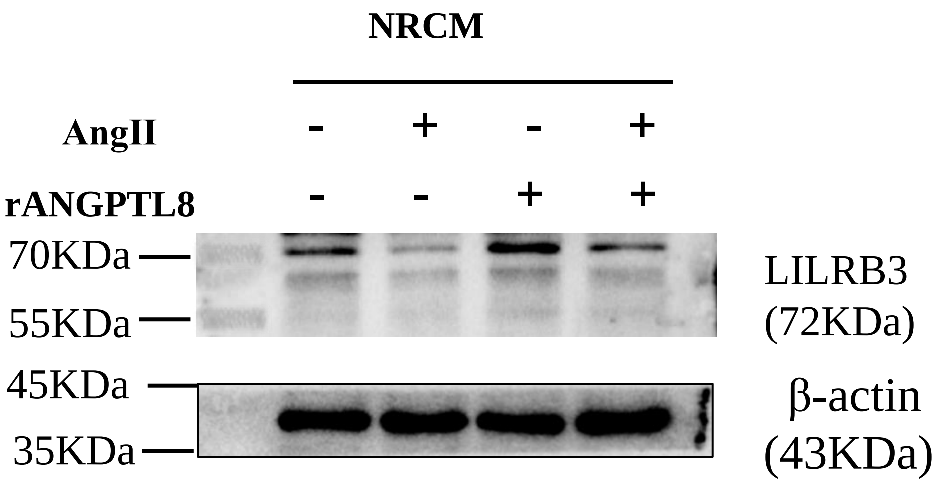

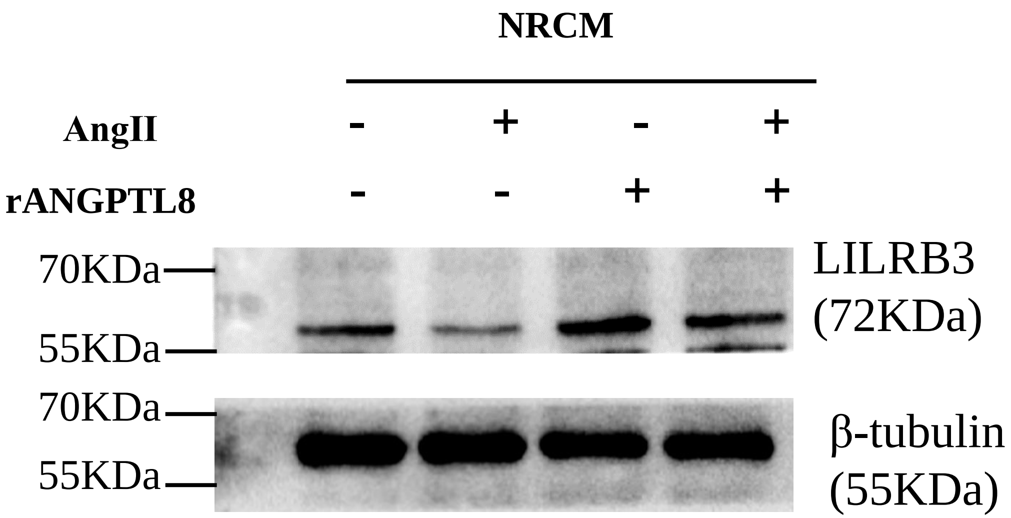

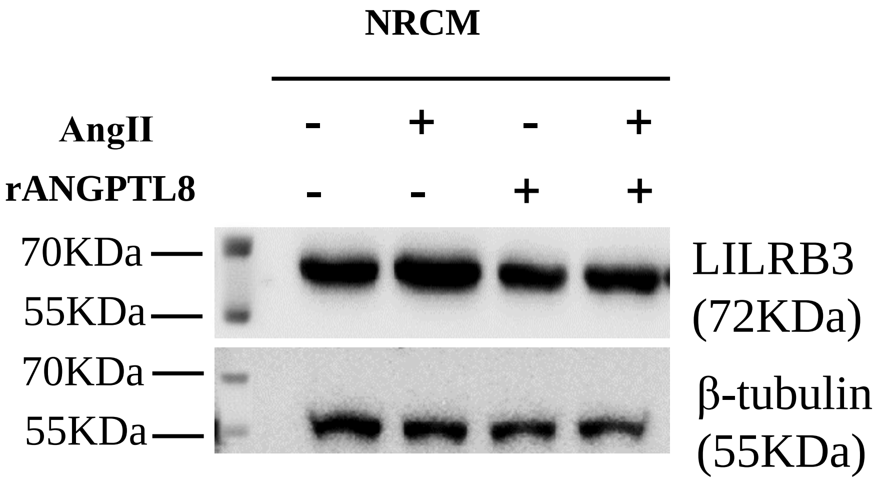

Original western Blots in Figure 7:

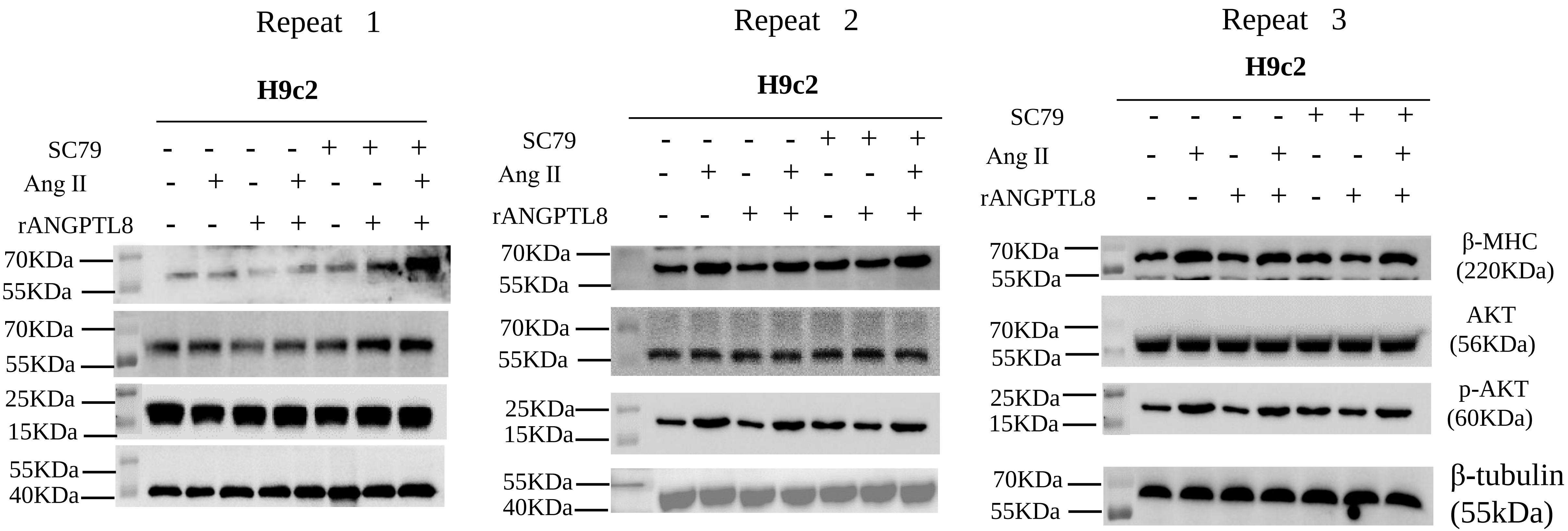

Original western Blots in Figure 8:

Repeat 2

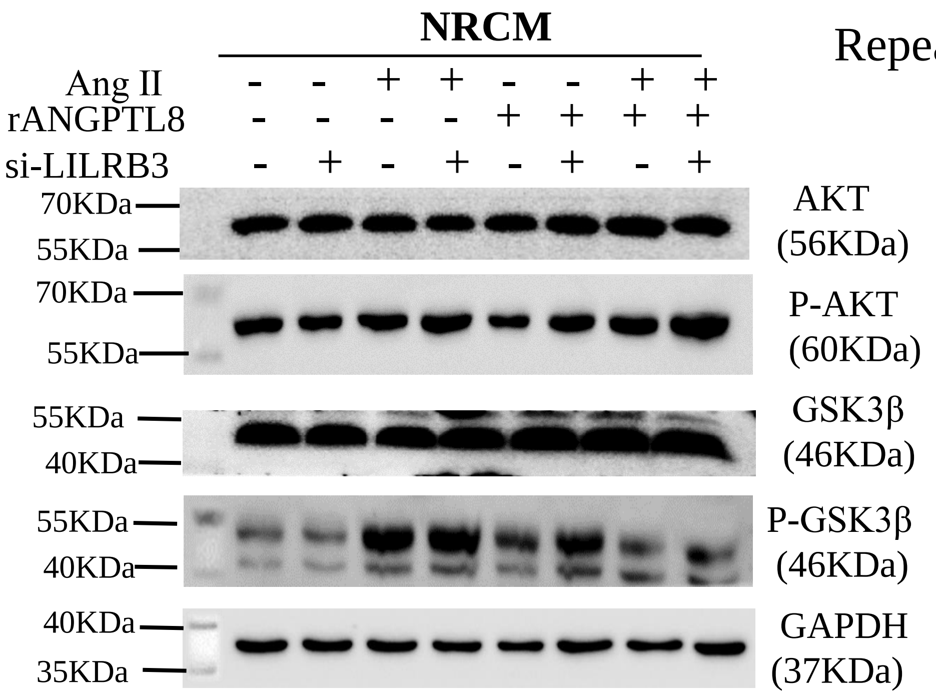

Repeat 1

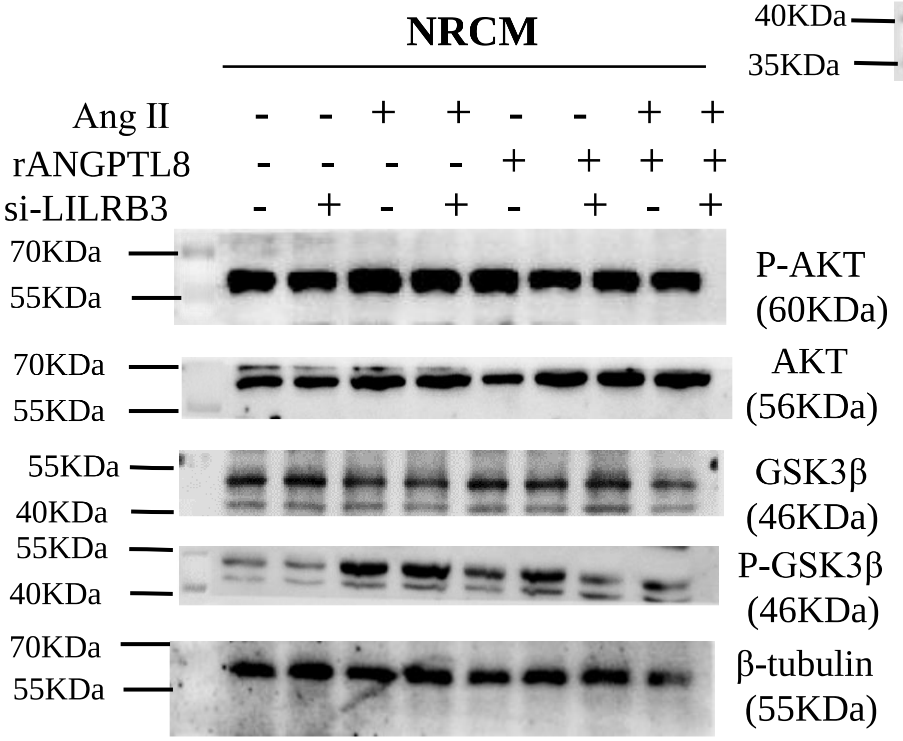

Repeat 3

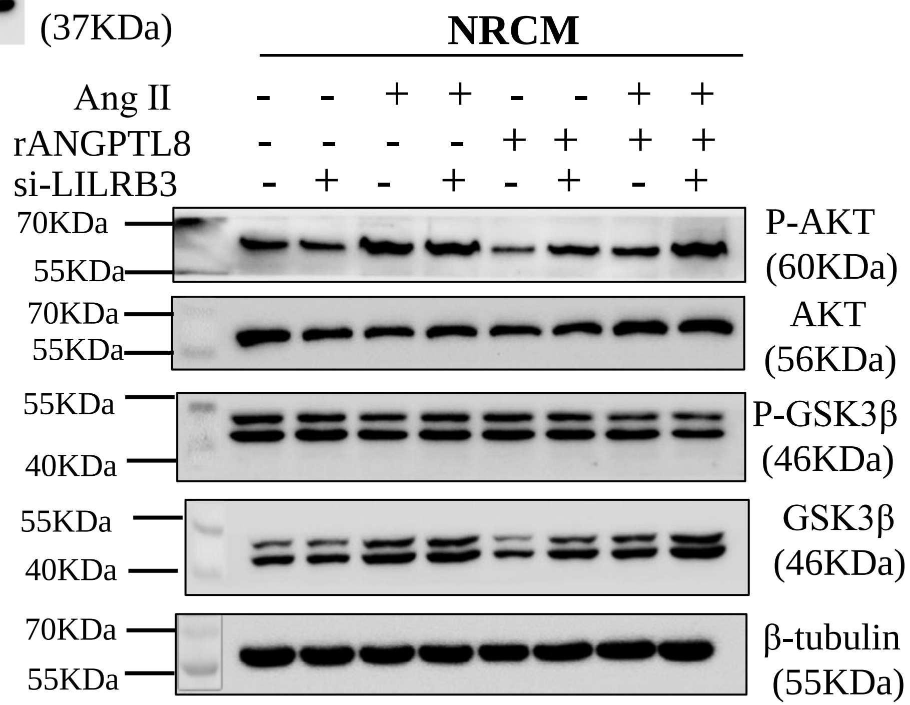

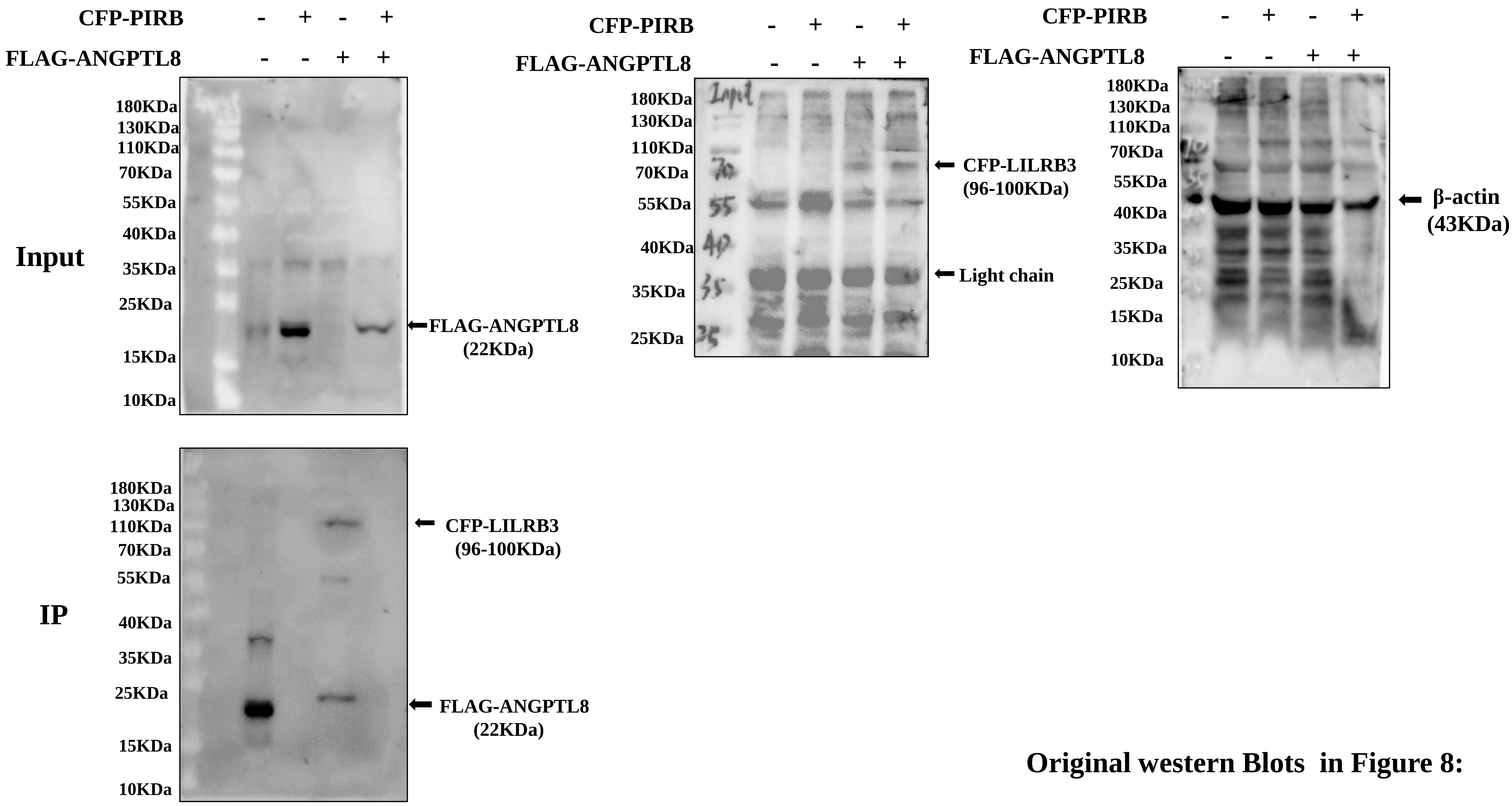

Original western Blots in Figure 8:
